# Design and Validation of a High-Throughput Reductive Catalytic Fractionation Method

**DOI:** 10.1021/jacsau.4c00126

**Published:** 2024-06-05

**Authors:** Jacob K. Kenny, Sasha R. Neefe, David G. Brandner, Michael L. Stone, Renee M. Happs, Ivan Kumaniaev, William P. Mounfield, Anne E. Harman-Ware, Katrien M. Devos, Thomas H. Pendergast, J. Will Medlin, Yuriy Román-Leshkov, Gregg T. Beckham

**Affiliations:** † Renewable Resources and Enabling Sciences Center, National Renewable Energy Laboratory, Golden, Colorado 80401, United States; ‡ Department of Chemical and Biological Engineering, University of Colorado, Boulder, Colorado 80303, United States; § Center for Bioenergy Innovation, Oak Ridge, Tennessee 37830, United States; ∥ Department of Chemical Engineering, Massachusetts Institute of Technology, Cambridge, Massachusetts 02139, United States; ⊥ Department of Organic Chemistry, Stockholm University, Stockholm SE-106 91, Sweden; # Institute of Plant Breeding, Genetics and Genomics, University of Georgia, Athens, Georgia 30602, United States; ∇ Department of Crop and Soil Sciences, University of Georgia, Athens, Georgia 30602, United States; ○ Department of Plant Biology, University of Georgia, Athens, Georgia 30602, United States

**Keywords:** lignin valorization, lignin-first biorefining, high-throughput reaction testing, high-throughput analysis, switchgrass

## Abstract

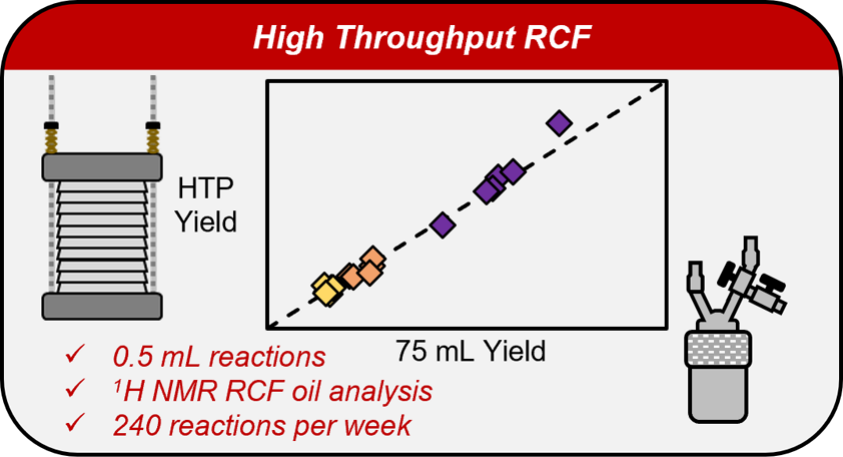

Reductive catalytic fractionation (RCF) is a promising method to extract and depolymerize lignin from biomass, and bench-scale studies have enabled considerable progress in the past decade. RCF experiments are typically conducted in pressurized batch reactors with volumes ranging between 50 and 1000 mL, limiting the throughput of these experiments to one to six reactions per day for an individual researcher. Here, we report a high-throughput RCF (HTP-RCF) method in which batch RCF reactions are conducted in 1 mL wells machined directly into Hastelloy reactor plates. The plate reactors can seal high pressures produced by organic solvents by vertically stacking multiple reactor plates, leading to a compact and modular system capable of performing 240 reactions per experiment. Using this setup, we screened solvent mixtures and catalyst loadings for hydrogen-free RCF using 50 mg poplar and 0.5 mL reaction solvent. The system of 1:1 isopropanol/methanol showed optimal monomer yields and selectivity to 4-propyl substituted monomers, and validation reactions using 75 mL batch reactors produced identical monomer yields. To accommodate the low material loadings, we then developed a workup procedure for parallel filtration, washing, and drying of samples and a ^1^H nuclear magnetic resonance spectroscopy method to measure the RCF oil yield without performing liquid–liquid extraction. As a demonstration of this experimental pipeline, 50 unique switchgrass samples were screened in RCF reactions in the HTP-RCF system, revealing a wide range of monomer yields (21–36%), S/G ratios (0.41–0.93), and oil yields (40–75%). These results were successfully validated by repeating RCF reactions in 75 mL batch reactors for a subset of samples. We anticipate that this approach can be used to rapidly screen substrates, catalysts, and reaction conditions in high-pressure batch reactions with higher throughput than standard batch reactors.

## Introduction

Reductive catalytic fractionation (RCF) is a lignin-first biorefining strategy that solvolytically extracts lignin from solid biomass in the presence of a heterogeneous catalyst to stabilize reactive intermediates into phenolic monomers.^1−4^ In the past decade, the scope of RCF processes has expanded to encompass diverse methodologies employing various solvents,^5−9^ flow conditions,^10−14^ hydrogen donors,^15−19^ catalysts,^20−23^ and feedstocks.^24−28^ These extensive studies have revealed common factors underlying the extraction and stabilization phenomenon, solidifying RCF as a repeatable and accessible process for fractionating biomass. Technoeconomic analysis and life cycle assessment have shown that RCF may be industrially viable for the production of aromatic chemicals,^29−32^ which can be upgraded to fuels,^33,34^ adhesives,^35^ and plastics,^36,37^ among others.^38,39^ Given its ability to selectively cleave aryl-ether linkages in lignin, RCF has also been used as an analytical method to study lignin structure, similar to thioacidolysis.^40,41^ Theoretical yields of phenolic monomers, the quantity of which reflect the aryl-ether content of the native lignin, can be routinely obtained from intact biomass.^26,28,42^ While typical RCF conditions do not lead to C–C bond scission, the cleavage of β–O–4 bonds also yields larger oligomers (dimers, trimers, etc.) that can be similarly related to the native lignin structure, further expanding the analytical value of RCF.^41,43−45^

Despite the reliability of RCF, gaining a deeper understanding of the lignin extraction and monomer formation processes under process-relevant conditions has proven difficult, in some part due to the low throughput of conventional bench-scale RCF. The reaction setup and postreaction workup to isolate the desired components for analysis can require multiple hours of researcher time per reactor, involving filtration and often multiple steps of rotary evaporation and liquid–liquid extraction (see the Results section for a full description of typical batch RCF procedure). Reactions are often conducted in pressurized batch reactors (50–1000 mL) where catalyst and biomass are intermixed. Solvent volumes are typically between 15 and 300 mL; however, substrate loading—and thus the solvent/biomass ratio—varies widely.^8^ Although reactions can be run at biomass concentrations as low as 10 g/L, high biomass/solvent ratios are critical to biorefinery economics,^31^ and increasing this ratio can impact monomer yield.^16^ Thus, realistic conditions require a substantial amount of biomass (1–3 g). Correspondingly, high catalyst loadings (10–20 wt % relative to the biomass loading) are needed because the required stabilization rate for maximum monomer production is directly linked to the biomass loading.^23,24,26,46^ For simplicity, researchers often perform reactions using long residence times with excess catalyst to ensure maximum lignin extraction and conversion to monomers. Aside from the limitation imposed by aryl-ether linkage abundance, the monomer yield at a particular residence time is subject to the effects that temperature, solvent, catalyst, and hydrogen donor have on the rates of lignin extraction, condensation, and stabilization in solution. The interdependence of these reaction parameters has also been demonstrated,^12,24,47^ further expanding the variable space that needs to be explored. Considering the experimental work needed to explore the expanding combinations of solvents, catalysts, and other modifications to typical RCF schemes, the throughput of conventional RCF reactions is limited.

High-throughput (HTP) experimental systems are advantageous for rapidly testing variables of complex systems across many research disciplines.^48−50^ Within biomass research, HTP methods have accelerated discoveries of plant composition and structure.^51^ These workflows typically proceed via biomass deconstruction followed by analysis of known products through methods such as pyrolysis–molecular beam mass spectrometry, acid hydrolysis coupled with high-performance liquid chromatography (HPLC) or ^1^H nuclear magnetic resonance (NMR) spectroscopy, and thioacidolysis.^52−55^ In several studies, liquid-phase HTP reactor systems were designed to analyze how pretreatment conditions and feedstock composition impact carbohydrate yields.^56−60^ In one design, modular 96-well plate reactors were sealed by stacking the plates together vertically, achieving 960 individual biomass pretreatment experiments in parallel with a stack of 10 plates.^57^ These reactors were developed without the ability to stir reactions or dose gases but achieved much higher throughput to enable the screening of entire populations of naturally variant poplar genotypes. Ultimately, many of these HTP approaches enabled heritable variation to be linked to measurable phenotypes through genomewide association studies.^61^ The adaptation of RCF to an HTP method could enhance the efficiency with which researchers could screen reaction parameters and open up RCF to data science methodologies currently unavailable due to low throughput.

Here, we report a method for conducting high-throughput RCF (HTP-RCF) reactions at 0.5 mL scale. The necessary developments to enable HTP-RCF included the tuning of catalyst loading and solvent composition under hydrogen-free (H_2_-free) conditions, the validation of a ^1^H NMR spectroscopy lignin quantification method, and the design of a streamlined reaction setup and workup protocol that improve throughput and minimize variability (including solids dispensing, product recovery, filtering, drying, and analytical preparation). The method was used to perform RCF reactions on 50 naturally variant switchgrass samples, demonstrating significant variation in monomer yield, oil yield, and S/G ratio across the sample set. Six samples were chosen for validation by repeating RCF in 75 mL batch reactors, and nearly identical results were obtained. The HTP method provides an approximately 15× increase in sample throughput and 26–60× reduction in catalyst, solvent, and biomass use, allowing for the rapid screening of RCF process parameters.

## Results

**Figure 1:**
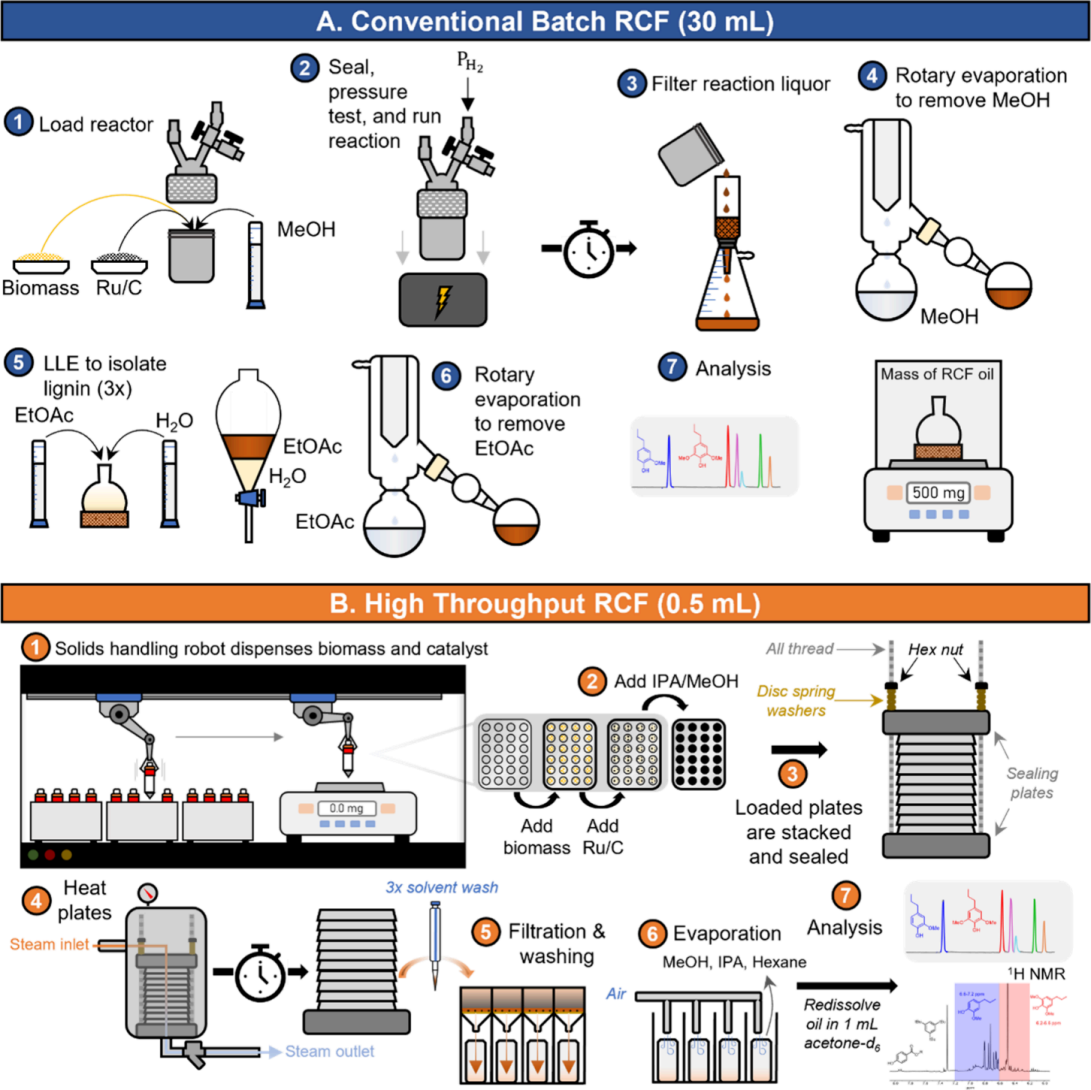
Comparison of procedures for reductive catalytic fractionation reactions at different scales. (A) Batch RCF conducted in a 75 mL reactor requires individual loading of high-pressure reactors. (1) Reactor is loaded with catalyst (e.g. Ru/C), biomass, and solvent (e.g. Methanol (MeOH)). (2) Reactor is sealed, pressurized, and heated for the desired reaction time. (3) Postreaction, the reaction mixture is filtered. (4) The solvent is evaporated from the filtered mixture in a rotary evaporator. (5) Ethyl acetate (EtOAc) and water are added to perform liquid–liquid extraction, and the aqueous layer is washed with two additional fractions of ethyl acetate. (6) The combined ethyl acetate fractions are evaporated to yield the RCF oil. (7) The RCF oil is massed to measure delignification, and the aromatic monomers are measured via chromatography. (B) HTP procedure described in this work. (1) Plates (affixed with O-rings) are loaded with biomass and catalyst with a solids-handling robot. (2) The reaction solvent (methanol, isopropanol (IPA) mixture) is added. (3) Plates are stacked and compressed between end plates with threaded rods to seal the wells. (4) Plates are heated (in a larger reactor with steam or in an oven in this work) for the desired reaction time. (5) Reaction mixtures from wells are filtered and washed using filter plates. (6) The collected reaction mixture and washes are evaporated under flowing air. (7) The oil is redissolved in acetone-*d*_6_ and subjected to analysis with ^1^H NMR spectroscopy for delignification and gas chromatography with flame ionization detector (GC-FID) for monomer quantification using a low-thermal mass column.

Figure 1A summarizes a typical batch RCF reaction protocol. First, the reactor is loaded with biomass, catalyst, and solvent, and the reactor is pressurized with hydrogen after purging with an inert gas. The reactors are then heated to the reaction temperature. After the desired reaction time, the reactor is cooled, and the mixture is filtered to separate the liquor from solid pulp and catalyst. The solvent is removed via evaporation to yield a crude RCF oil that is subjected to liquid–liquid extraction to isolate the lignin-rich RCF oil (organic layer) from extracted carbohydrates (aqueous layer). The combined organic layers are evaporated to recover the RCF oil, which is massed and analyzed for monomer content. The commercial availability of multireactor systems allows researchers to operate multiple reactions in parallel, thus shifting the bottleneck to the postreaction workup. This limits the throughput of a single researcher to approximately six reactions per 8 h of work depending on the duration of the reaction, although the workup may be split into multiple days for convenience.

To enable HTP-RCF, substantial modifications to the typical RCF procedure were needed. Given the need for low substrate loadings to limit material usage, it is necessary to also reduce solvent loadings to maintain the appropriate solvent-to-biomass ratios. This, in turn, requires a decrease in reactor volumes. Although mini-scale reactors could be feasible, the operation of RCF in separate reactors requires individual attention for sealing, heating setup, and reactor quenching for each reactor. We began this study by demonstrating the feasibility and utility of small-scale RCF (0.5 mL solvent) using a custom plate reactor. Aside from the issue of scale, direct application of the conventional batch RCF protocol to small-scale reactions did not increase throughput because a substantial amount of researcher time is dedicated to the postreaction workup procedure. Thus, in the sections that follow, we then present adaptations to the pre- and postreaction procedure to increase throughput. The resulting, optimized procedure is shown in Figure 1B.

### Design of an HTP Plate Reactor

**Figure 2:**
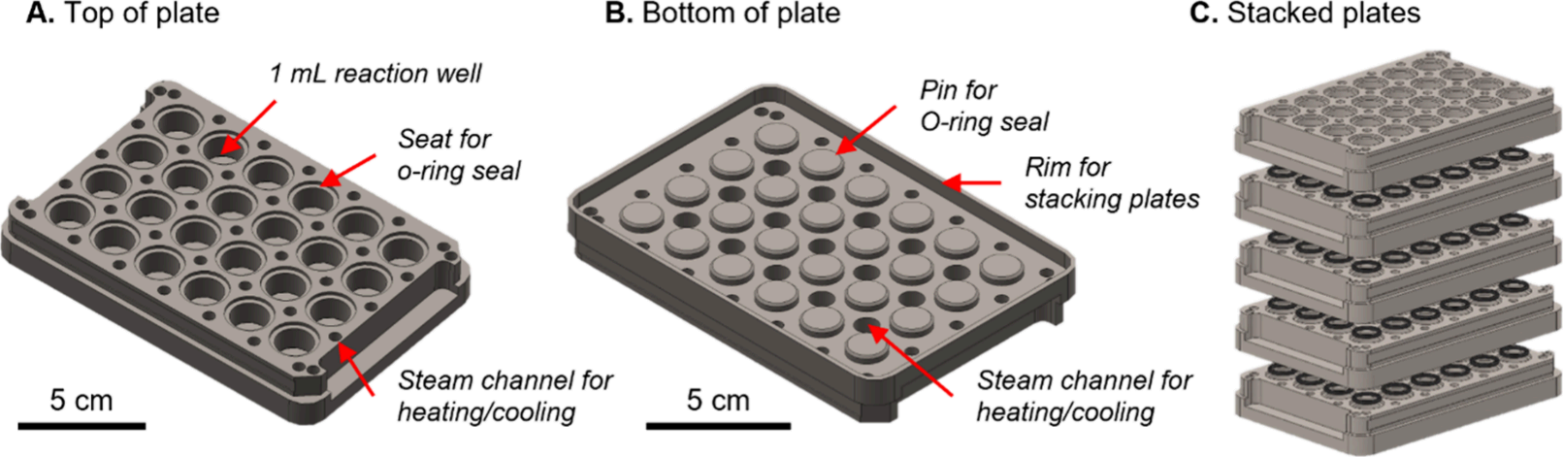
Design of plate reactors. (A) Top view of plate reactor showing 1 mL reaction wells, O-ring seats, and steam channels for heating/cooling. (B) Bottom of plate with pins for sealing the plate directly below. (C) Stack of five plates with O-rings shown.

RCF reactions are often conducted using organic solvents such as methanol (MeOH) at temperatures well above their boiling point, and thus reaction pressures can exceed 80 bar depending on the solvent, temperature, and external gas pressure.^47^ To safely contain the pressure, researchers typically use commercially available, high-pressure batch reactors,^4^ although mini-reactors formed from Swagelok unions have been used successfully with lower reaction volumes (5–10 mL).^38,62,63^ Inspired by previous work on a 96-well plate design,^57^ we designed stackable reactor plates containing 24 wells in a 4 × 6 arrangement (Figure 2). To individually seal each reaction well, pins machined into the bottom of each plate were affixed with an O-ring that fits into a groove around corresponding reactor wells of the plate directly below. The plates can then be stacked and compressed between end plates using threaded rods and disc spring washers, which thereby compress the O-ring and provide the necessary seal (the Supporting Information (SI) includes the reactor design drawings; see Figure S1A–F, Table S1 for equipment list). In this way, the space between the wells is open to the heating medium (air or steam, *vide infra*), which in conjunction with the hollow machined channels provides improved heat transfer. Furthermore, each well is a closed system, and headspace is not shared between wells. In the event of a leak, the solvent vapor would leak into the interplate space, which would mix with the heating medium and thus not contaminate other wells (Figure S1F).

To verify that each well could maintain reaction pressure, solvent recovery experiments were conducted by loading 500 mg of water into each well in a 24-well plate, heating to a specified temperature, and then measuring the mass of water remaining after a desired time. When water was heated to 225 °C for 3 h, the average mass recovery was 93 ± 2% across 24 wells, indicating a solvent loss rate of ∼12 mg/h (Figure S2A). Increasing the temperature to 250 °C, which is the maximum temperature that RCF is typically conducted, average recoveries of 94 ± 2 and 84 ± 4% were measured for 1 and 3 h experiments, respectively, indicating an average loss of approximately 30 mg water per hour (Figure S2B,C). The increase in the leakage rate with temperature is likely due to the increase in water vapor pressure (26 bar at 225 °C relative to 38 bar at 250 °C) but also may be affected by the increased plasticity of the PTFE O-ring. The average recovery of outer wells (*n* = 16) was not significantly different to the recovery of internal wells (*n* = 8) for all experiments, indicating that edge effects are negligible (α = 0.05, two-sided *t* test with unequal variance; *p* value (1 h, 225 °C) = 0.39; *p* value (1 h, 250 °C) = 0.17; *p* value (3 h, 250 °C) = 0.68). Whereas the maximum pressure is dictated by the solvent used, the HTP reactor can operate at 225 °C for 3 h at ∼40 bar, which is typically sufficient for lignin extraction to reach near theoretical limits.^19^ Higher temperature operation near 250 °C should likely be limited to only shorter (∼1 h) reaction times to avoid the impacts of solvent loss.

### Reaction Engineering at 0.5 mL Scale

With a viable plate reactor system in hand, we next turned to the operation and analysis of RCF reactions at the 0.5 mL scale. When designing HTP reaction systems, ideally all aspects from larger-scale reactions can be translated to the smaller scale. However, several aspects of conventional RCF are not feasible in the stacked plate design. First, the reactor wells are not stirred, and thus, mixing occurs only via convection upon solvent heating. Although the lack of stirring may increase local extracted lignin concentrations,^64^ stirring rate was previously shown to weakly affect total monomer yield.^33,65^ The second limitation is the lack of high-pressure H_2_ gas dosing. Although exogenous H_2_ pressure increases the monomer formation rate in catalyst-limited conditions,^66,67^ H_2_-free RCF schemes capable of obtaining monomer yields near theoretical limits have been reported.^16,17,20^ In addition to its relevance to realizing an industrially viable RCF process,^31^ H_2_-free RCF serves as an important model experiment for the HTP method given the inability to dose high-pressure gases because a sufficiently high stabilization rate is needed to study extraction-limited, substrate-dependent behavior.^66^ Previous studies have also highlighted that monomer yields from H_2_-free RCF are subject to interrelated effects of both catalyst and solvent on the stabilization rate.^47,68,69^ Based on the work from Rinaldi et al. in which higher monomer yields were obtained during H_2_-free RCF in IPA compared to MeOH,^69^ we hypothesized that the addition of IPA to the MeOH solvent could increase the rate of H_2_-free stabilization while retaining a high degree of lignin extraction. Beneficially, IPA also exhibits a lower vapor pressure compared to MeOH (IPA: 26 bar,^70^ MeOH: 38 bar^71^ at 200 °C), thus reducing the reactor pressure and the chance of well leakage. We chose Ru/C as the catalyst due to its common use in the RCF literature, commercial availability, and lower cost than Pd/C and Pt/C; however, the yield and selectivity results obtained are likely dependent on this choice.^19^

**Figure 3:**
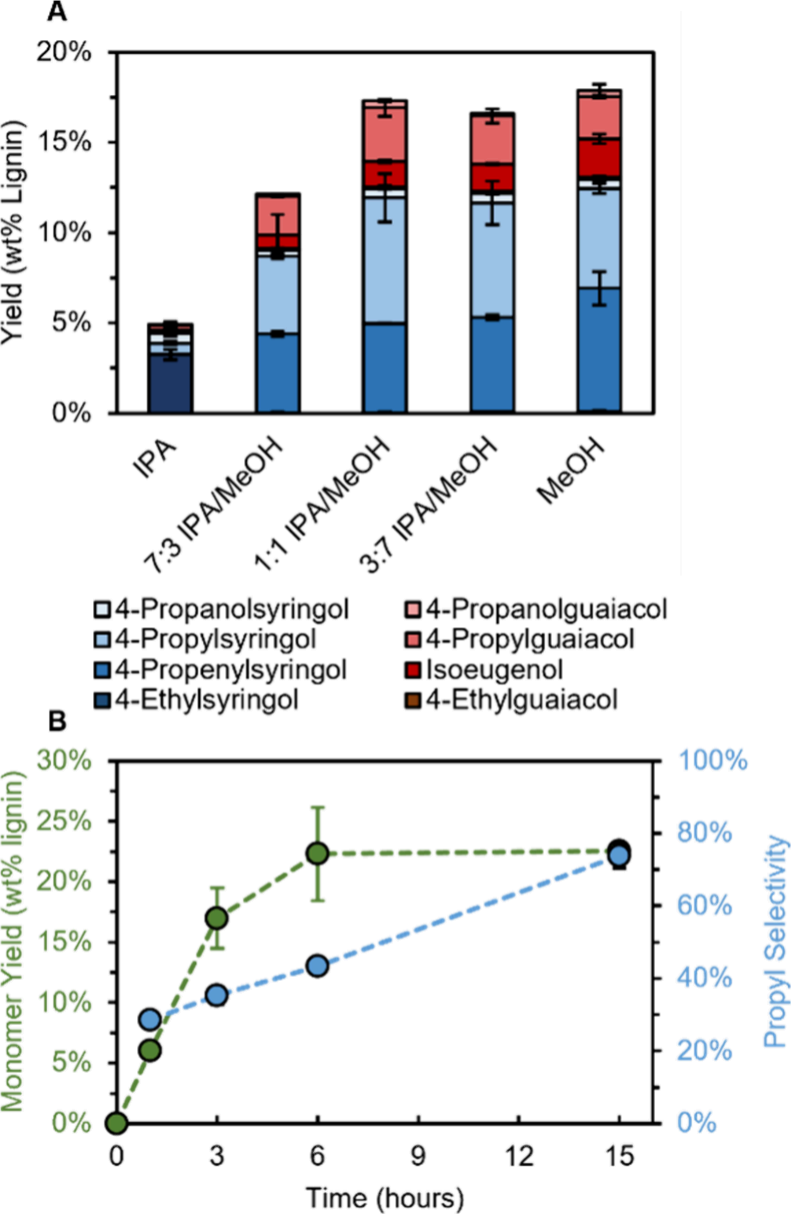
Impact of solvent composition and residence time in HTP RCF. (A) Comparison of monomer yields from reactions using IPA/MeOH mixture solvents showing that 1:1 (v/v) IPA/MeOH can achieve similar monomer yields compared to MeOH while retaining higher selectivity to monomers with propyl side chains. Conditions: 50 mg poplar, 10 mg of 5 wt % Ru/C, 0.5 mL solvent, 15 h at 180 °C, via the “Workup I” method. Error bars are the range between duplicate measurements. (B) Time course reactions demonstrating similar yields for 6 and 15 h reactions. Conditions: 50 mg poplar, 10 mg of 5 wt % Ru/C, 0.5 mL solvent, 1–15 h at 200 °C, via the “Workup I” method. Error bars are the standard deviation of triplicate measurements. Tables S3 and S4 provide the quantitative data in this figure.

RCF reactions were performed with 50 mg of poplar and 10 mg Ru/C by placing the loaded plate reactors in a preheated (static) oven at 180 °C for 15 h (see Table S2 for compositional analysis of poplar substrate used for exploratory experiments). Varying ratios of IPA/MeOH were used while keeping the solvent volume constant at 0.5 mL (Figure 3A, Table S3), and the aromatic monomer yield was measured via GC-FID (see the Supporting Information). We note that this workup for these reactions (referred to as Workup I) was not yet optimized for maximum throughput, as the focus was first to analyze the reactivity in the plate reactors (see Supporting Information S1.3 for workup details). The total yield of aromatic monomers was consistent at ∼17% for reactions in MeOH (17.9 ± 1%), 3:7 IPA/MeOH (16.8 ± 2%), and 1:1 IPA/MeOH (17.3 ± 2%), indicating that similar yields can be obtained with 1:1 IPA/MeOH (v/v) compared to MeOH while reducing reactor pressure (Figure 3A andTable S3, ± indicates the range of duplicate reactions). Over this range, the selectivity of 4-propyl substituted monomers increased with IPA content from 44 ± 3% for MeOH to 57 ± 3% for 1:1 IPA/MeOH, whereas selectivity to 4-propenyl monomers decreased, indicating a higher hydrogenation rate. Increasing the IPA amount further to 7:3 IPA/MeOH and ultimately pure IPA resulted in a decreased total monomer yield, likely due to the poorer ability of IPA to extract lignin, which has been correlated to its polarity.^5,23^ Notably, reactions in IPA produced high yields of ethyl-substituted monomers (Figure 3A). Previous work on H_2_-free RCF had shown that this pathway may proceed through dehydrogenation of coniferyl and sinapyl alcohol intermediates followed by C–C bond scission, but this primarily occurred on Pd/C, and Ru/C showed almost no activity for this route.^19,20^

RCF conditions are typically chosen to maximize lignin extraction, which requires high temperatures, long reaction times, and/or the addition of water to the reaction solvent.^5,6^ To enhance extraction rate, the reaction temperature was increased to 200 °C using 1:1 IPA/MeOH. The total monomer yield and the selectivity to 4-propyl products increased relative to reactions performed at 180 °C to 22.5 ± 0.7 and 74 ± 3%, respectively, for reactions run for 15 h in 1:1 IPA/MeOH (Figure 3B, Table S4, ± indicates the standard deviation of triplicate reactions). Reactions were then run using the same loadings for shorter reaction times with the goal of increasing throughput. The total monomer yield continued to increase between 1 and 6 h but remained constant from 6 to 15 h, indicating that 6 h reactions can provide similar information as the 15 h reactions. Selectivity to 4-propyl products continued to increase during this time, however, from 43.3 ± 7% at 6 h to 74 ± 3% at 15 h (Figure 3B).

**Figure 4:**
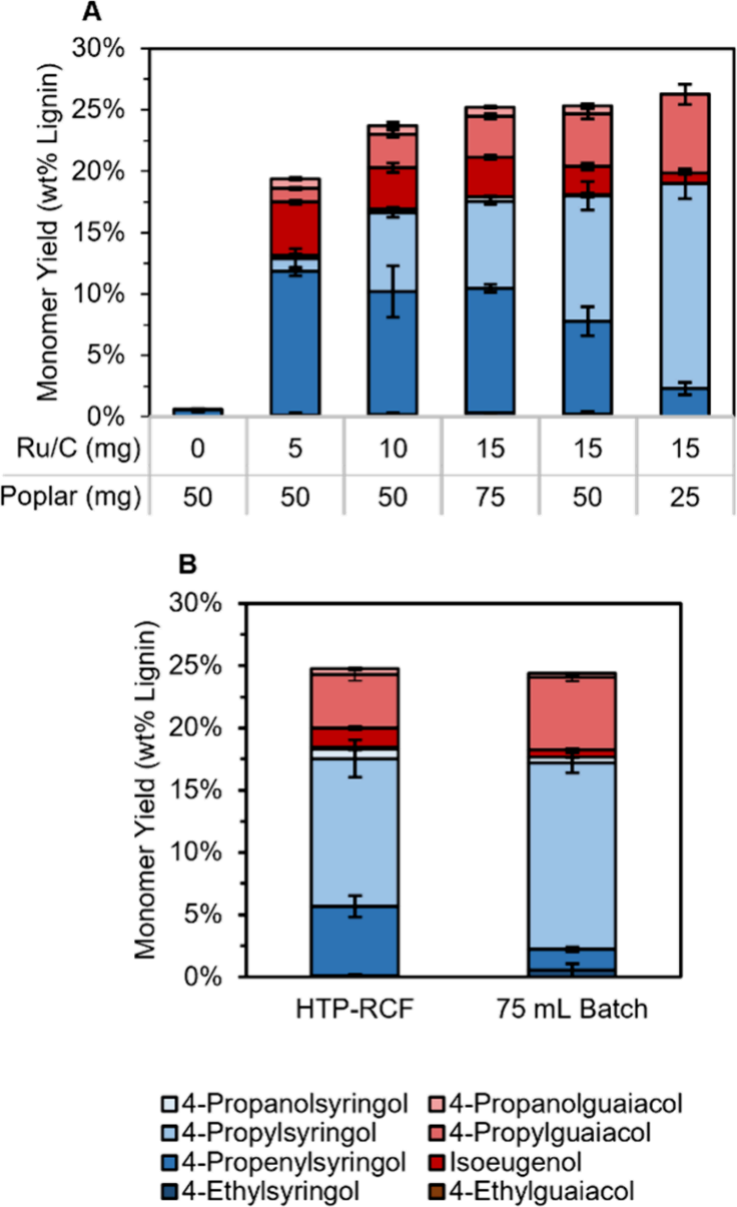
Effect of catalyst loading on monomer yield and comparison to 75 mL reactors. (A) Monomer yields from HTP reactions conducted with varying catalyst wt % loading (mass catalyst/mass poplar). Conditions: 25–75 mg poplar, 0–15 mg Ru/C, 200 °C, 0.5 mL 1:1 IPA/MeOH, 6 h, via the workup “Method II” (*vide infra*). Error bars are the standard deviation of 12 measurements. (B) Comparison of HTP-RCF reaction to an analogous reaction in a 75 mL batch reactor. HTP conditions: 50 mg poplar, 15 mg 5 wt % Ru/C, 0.5 mL 1:1 IPA/MeOH, 6 h, 200 °C. 75 mL batch conditions: 2 g poplar, 600 mg 5 wt % Ru/C, 20 mL 1:1 IPA/MeOH, 6 h, 200 °C. Error bars represent the standard deviation of four (HTP-RCF) and three (75 mL batch) measurements. Tables S5 and S6 provide the quantitative data shown in this figure.

Previous work has identified two major reaction regimes of RCF measured by monomer yields; namely, at low catalyst loadings or hydrogen pressures, monomer yields are limited by the catalytic stabilization rate. Conversely, at more forcing catalytic conditions where aryl-ether linkages are quantitatively converted to aromatic monomers, yields are instead governed by the solvolytic lignin extraction rate.^66^ Proper selection of operating conditions is thus important to ensure that reaction results (RCF monomer and oil yields) reflect the desired information, such as catalyst activity or biomass variability. Batch reactions are typically conducted with sufficient catalyst (10–20 wt %, reported as the catalyst mass relative to the biomass substrate mass loading) so that reactions are limited by the rate of lignin extraction.^66^ Given the significantly different reaction volumes and the lack of stirring, we deemed it important to examine the operating regimes encountered in the plate reactors. We thus performed reactions with catalyst loadings varying from 0 to 60 wt % Ru/C (Figure 4A, Table S5).

As expected, reactions without catalyst yielded only 1.0 ± 0.2% monomers. Upon the addition of 10 wt % catalyst, the yield increased to 19.4 ± 0.6%. As catalyst loading increased to 60 wt %, the monomer yield plateaued at 26.3 ± 2%. Above 20 wt %, the total monomer yield was only a weak function of catalyst loading, and extraction-limited conditions were obtained. However, selectivity to 4-propyl over 4-propenyl substituted products still increased with increasing catalyst loading throughout the studied range (Figure 4A, ± indicates the standard deviation of 12 reactions). The higher weight percent of catalyst needed to reach extraction-limited conditions compared to previous reports may be due to the lack of stirring, leading to insufficient mixing. Conversely, catalytic stabilization activity can be analyzed where the total monomer yield is a function of the catalyst loading (around 10 wt %).

To validate the results above, we performed an analogous unstirred reaction in a 75 mL batch reactor (20 mL 1:1 IPA/MeOH) with identical solvent/biomass (0.1 g/mL) and catalyst/biomass (30 wt %) loadings. This reaction yielded nearly identical results compared to the plate reactors in terms of total monomer yield (75 mL: 24 ± 1%, plates: 24.7 ± 2, *p* value from two-tailed, unequal variance *t* test = 0.78,) and S/G ratio (75 mL: 2.23 ± 0.04, plates: 2.42 ± 0.09, *p* value = 0.02), demonstrating that the HTP reactor accurately recovered results from the 75 mL reactor scale (Figure 4B, Table S6). Selectivity to 4-propenyl monomers was higher in the HTP-RCF reactions, indicating a lower rate of hydrogenation. Although the underlying reason for this is not clear, it could have resulted from the difference in heating rate from the electrically heated batch multireactor system compared to the HTP-RCF system heated by the static oven or steam. Alternatively, variation in reactor geometry could affect the mixing of the catalyst, solvent, and in situ generated gas.

### Measurement of Delignification

In addition to the aromatic monomer yield, RCF practitioners often measure the total amount of extracted lignin, referred to as delignification or RCF oil yield. However, given the low mass of substrate, direct gravimetric measurement of the extracted oil, as is typically done for larger-scale RCF experiments, was infeasible. Specifically, the gravimetric oil mass (5–10 mg oil from 50 mg total biomass) was overestimated and highly variable at this scale even when identical wells were combined and processed together to increase the measured mass (Tables S3 and S4).

**Figure 5:**
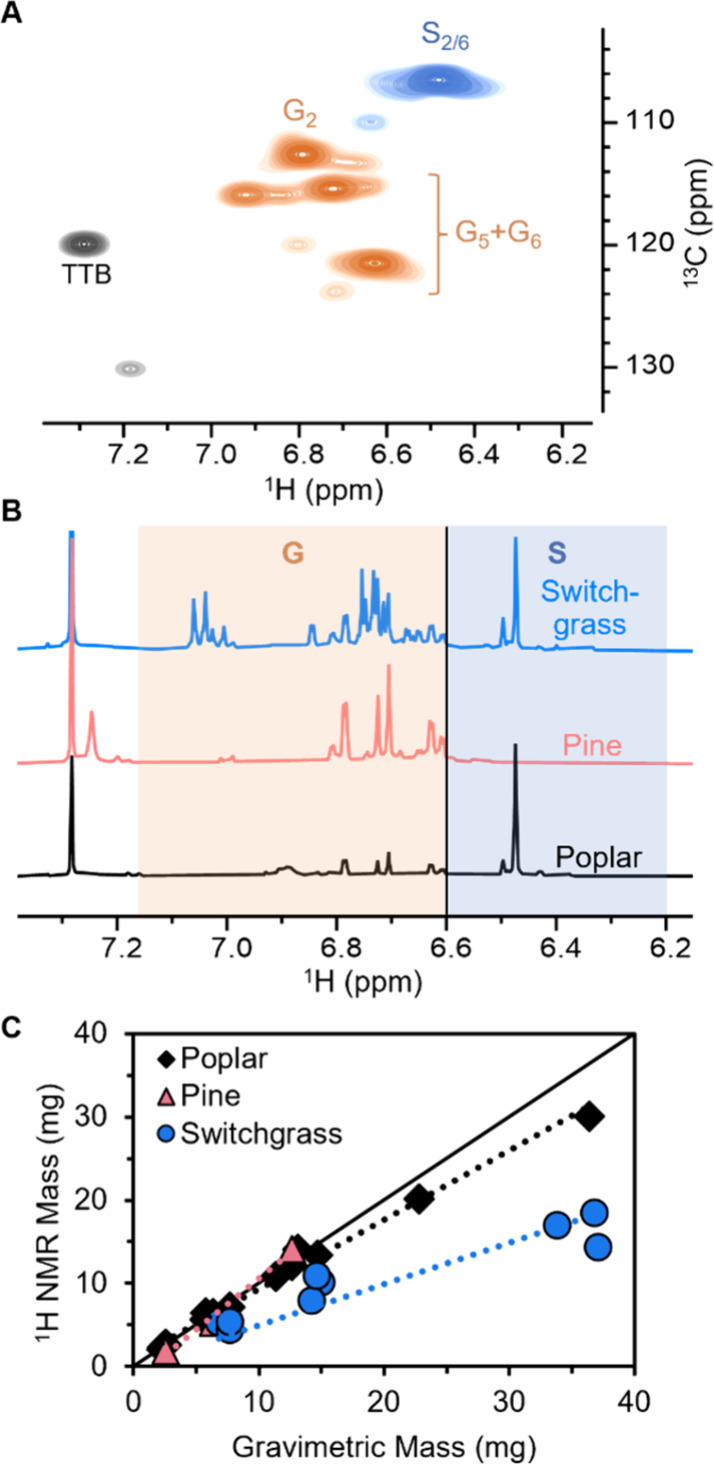
Development of ^1^H NMR for RCF oil quantification. (A) ^1^H–^13^C HSQC NMR spectrum of poplar RCF oil showing separation between syringyl and guaiacyl resonances. 1,3,5-tri-*tert*-butylbenzene (TTB) is used as the internal standard. (B) ^1^H NMR spectra of RCF oil from various substrates with the syringyl region highlighted in blue and the guaiacyl region highlighted in orange. (C) Agreement between the gravimetric (*x* axis) and ^1^H NMR measured (*y* axis) RCF oil mass for poplar (five substrates), pine (one substrate), and switchgrasses (three substrates) for various concentrations of oil. Calibration was done by minimizing the percent error for poplar values. In all cases, RCF oil was generated in 75 mL batch reaction using 2 g biomass, 400 mg Ru/C, 30 mL MeOH, 3 h, 225 °C, ethyl acetate/water liquid–liquid extraction. Table S7 provides ^1^H NMR shifts used, and Table S8 provides quantitative information from panel C.

To overcome the difficulty encountered in gravimetric measurements, we sought to develop a suitable NMR spectroscopy-based method to quantify extracted lignin from small quantities of RCF oil. Although quantitative information can be obtained in minutes from a routine ^1^H NMR experiment, more complex and therefore time-consuming and/or nonquantitative NMR experiments are typically preferred for lignin analysis due to its structural complexity leading to overlapping resonances.^72,73^ Fortunately, ^1^H–^13^C heteronuclear single quantum coherence (HSQC) NMR spectroscopy reveals that the aromatic proton resonances overlap substantially less in RCF oil than in the corresponding native biomass.^17^ In the HSQC spectrum of RCF oil from poplar (Figure 5A), the S_2/6_ resonances are centered at 6.45 ppm, whereas the three guaiacyl resonances are centered at 6.63, 6.74, and 6.80 ppm. Previously, Samec et al. utilized these characteristic shifts to determine the yield of S- and G-type aromatics in birch RCF oil using a ^1^H NMR method.^11^ We sought to expand on this methodology and explore its potential use as a replacement for the gravimetric oil yield.

To probe the viability of the proposed method, the ^1^H NMR spectra of poplar, pine, and switchgrass RCF oil were compared to model compounds representative of lignin structures (Figure 5B, Figure S3, Table S7). RCF reactions were performed in 75 mL batch reactors with 2 g of biomass, 400 mg Ru/C, and 30 mL MeOH for 3 h, and an ethyl acetate/water liquid–liquid extraction was performed to isolate the RCF oil (see Supporting Information S1.1 for reaction procedure). In poplar and switchgrass, which contain both S and G lignin, aromatic resonances ranged from ∼6.2 to 7.0 ppm, whereas resonances in the spectrum of pine RCF oil, which contains only G lignin, were mostly confined downfield of 6.6 ppm. In accordance with the model compound spectra, these data indicate that S- and G-type functionality could potentially be distinguished by their resonance positions upfield and downfield of 6.6 ppm, respectively. Exceptions to this observation were encountered, including the S_2/6_ resonance of 4-propenylsyringol (6.67 ppm) and the alpha proton of unsaturated (4-propenyl) side chains (6.32 ppm) (Figure S3B).

In addition to the S, G, and H monolignols, lignins can also exhibit aromatic ester-linked units, such as *p*-hydroxybenzoic acid (*p*-HBA), which esterifies S units in some hardwoods, and *p*-coumaric acid and ferulic acid, which are found in grasses such as switchgrass and corn stover.^26,28^ During the RCF process, these species can undergo further reactions such as esterification with the alcohol solvent, decarboxylation, and double bond hydrogenation for the hydroxycinnamates. *p*-HBA and its ester analogue, methyl paraben, exhibit distinct ^1^H NMR resonances located at 7.9 ppm, allowing for their direct measurement.^19^ From *p*-coumaric and ferulic acids, a total of 10 possible products must be considered (including *p*-coumaric acid and ferulic acid). Conveniently, most of these products exhibited unique resonances dispersed among G aromatic units in the ^1^H NMR spectrum (Figure S4). Products deriving from *p*-coumaric acid show characteristic doublets that are easily distinguished from the RCF oil. The most problematic product is dihydroferulic acid (3-(3-methoxy-4-hydroxyphenyl)-propionic acid), whose resonances overlap with lignin-derived G-type compounds. However, carboxylic acid groups are typically esterified in the presence of alcohol solvent with sufficient residence time.^19,26^ The methyl ester analogue, methylhydroferulate (methyl-3-(3-methoxy-4-hydroxyphenyl)-propanoate), shows a distinguishable resonance at approximately 6.88 ppm (Figure S4B).

The ^1^H NMR spectra of RCF oil contain clearly defined resonances arising from aromatic protons and appeared to be delineated broadly by S- and G-type functionality (Figure 5B). Nonetheless, use of aromatic resonances in the ^1^H NMR spectra for quantification of delignification requires careful consideration. Lignin extraction yields are typically reported on a mass basis (mass of lignin extracted relative to mass of lignin in the biomass initially loaded), but integration of the NMR spectrum gives a mole-based measurement of the corresponding protons. For translation to mass yields, the molar measurements must be multiplied by a molecular mass, which further requires identification of individual resonances corresponding to known compounds. Although many components of RCF oil are known, quantification of each species in RCF oil from a ^1^H NMR spectrum is infeasible due to low abundance.

To overcome this challenge, we posited that the aromatic integrals could be converted to the oil mass using a calibration factor. We note this is not the average molecular mass of species in the RCF oil, which includes larger oligomers, but rather the average mass of the aromatic unit with the accompanying side chain. ^1^H NMR spectra were recorded for samples with varying concentrations of RCF oil, and the obtained integrals were compared to the known gravimetric masses. Given the apparent distribution of S and G signals in the poplar, pine, and switchgrass spectra, this calibration value can be further informed via differentiation between these units because they give rise to a different number of protons per aromatic unit, although we note that this is not essential (*vide infra*). The optimal calibration values were found by manipulating the S unit mass (from which the G unit mass can be obtained by subtracting the mass of one CH_2_O group, assuming that the same side chain chemistry occurs for S and G units). An average S unit mass of 200 mg/mmol was obtained for poplar measurements (mean absolute error (MAE): 0.97 mg, average percent error: 6.8%), which is close to the molecular weight of 4-propylsyringol (196 mg/mmol) (Figure 5C, Figure S5). The poplar calibrated values were also satisfactory for calculation of oil mass from pine (MAE 1.0 mg, average percent error: 17.2%). Integration of the S region in pine quantifies a small number of aromatic units leading to a calculated S/G ratio of 0.19, indicating the presence of G-type species that nonetheless exhibit resonances in the S region.

Compared to poplar and pine samples, NMR measurements on switchgrass oils showed significant deviations from gravimetric values. The gravimetric oil mass was much greater than the calculated value using the poplar calibrated average aromatic unit mass (MAE: 8.8 mg, average percent error: 39.5%). Solving for a switchgrass specific value of the average aromatic unit mass gave a value of 320 mg/mmol, which is greater than any possible lignin unit and accompanying side chain mass. Although oil mass is a commonly employed metric and is important for process design, it does not guarantee the exclusive measurement of lignin aromatics. Nonlignin species derived from extractives, carbohydrates, or inorganic species may also be dissolved in the organic fraction and thus inflate the measured mass. Alternatively, decomposition of lignin products may also reduce the measured oil mass. Previous work from our group revealed a large mismatch between the gravimetric oil yield and actual delignification extent measured by compositional analysis for nonwoody substrates.^27^ In this case, the ^1^H NMR method is expected to reflect the lignin content in the oil more accurately than the mass of organic soluble oil; however, this claim needs further validation, such as by identification of the nonlignin species that can inflate the oil mass and extension to additional biomass sources.

Aside from the calibration of the S unit mass, an additional source of error can be traced to assigned S and G regions in the ^1^H NMR spectrum. The S/G ratios obtained from the ^1^H NMR method for poplar were systematically lower than those obtained from ^1^H–^13^C HSQC and from monomer products quantified via GC-FID (Figure S6). This incorrect measurement of S/G ratio causes underestimation in the delignification; however, the sensitivity of the calculated oil mass to the S/G ratio is still low. The error in NMR oil masses for poplar did not show a dependence on S/G ratio of the oil (Figure S7), and utilizing the monomer S/G ratio measured by GC-FID for calculation of oil yield for poplar only increased the calculated oil mass by 6.5% on average. The low sensitivity results from the low relative differences between S and G unit masses combined with the narrow range of S/G ratios encountered in naturally occurring biomasses (0–5). This indicates that even total aromatic protons (integral of 6.2–7.2 ppm) would also be an appropriate metric for delignification (for further consideration of this source of error, see Supporting Information S1.6.1). Overall, the ^1^H NMR method agrees well with gravimetric mass measurements and was thus deemed appropriate for HTP experimentation given the short time and small sample volume required for the measurement.

In addition to the total RCF oil, we observed that the resonances of S and G monomers were distinguishable from the surrounding oil resonances in each sample provided that they are present in sufficient amounts (∼3 wt %). Classification of the S monomers by their side chain substitution was possible because of the varying position singlet S_2/6_ proton (Figures S3B and S8). For G monomers, isoeugenol is uniquely identifiable by its resonance at 6.99 ppm; however, 4-ethylguaiacol, 4-propylguaiacol, and 4-propanol guaiacol all show some degree of overlap (Figures S3 and S8). Although the ^1^H NMR spectrum cannot provide unambiguous determination of G monomer identity, selectivity to ethyl substituted monomers is usually low for conventional RCF reactions. Furthermore, selectivity—and thus the likely abundance of 4-propyl/4-ethylguaiacol—can be inferred by the S region or from knowledge of reaction conditions. For the selection of oils presented here, the individual monomer yields calculated from their integrations were closely aligned with those measured with GC-FID (Figure S9). Alternative techniques such as HPLC and GC-FID are likely preferred because of their reliability and lower likelihood of overlapping peaks, but the ability to quantify aromatic monomers from a ^1^H NMR spectrum offers a powerful supplement to the RCF practitioners’ toolbox, either as a form of primary measurement for those who do not have access to the necessary analytical equipment or analytical standards or as an independent verification of the conventional chromatographic measurement methods.

### Development of the HTP-RCF Procedure

The results from exploratory HTP experiments showed that RCF reactions run at 0.5 mL scale in the plate reactors are representative of results obtained at larger scales. Although this alone can provide an increase in throughput, a significant amount of researcher time is required prereaction for reactor loading and postreaction for RCF oil isolation and analysis.

To increase throughput, we aimed to make further improvements to the procedure to minimize the time required per sample. First, we focused on the loading of solid biomass and catalyst in the reactor. For standard bench-scale experiments (e.g., 75 mL scale), quantitatively adding biomass and catalyst to the reactor is straightforward. However, manually loading small masses (5–75 mg) of solids into the reactor wells was tedious and error-prone because of transfer losses. To overcome this, the plate reactors were designed to be compatible with a solids-handling robot (Symyx Powdernium) that allowed for autonomous loading of catalyst and biomass from preloaded dispensing containers (hoppers) with online mass measurements to verify proper loadings (Figure S10). Precise dispensing from up to 200 hoppers (20–75 ± 1 mg biomass; 0–15 ± 1 mg Ru/C catalyst in this work) into ten 24-well plates was accomplished in less than 24 h without supervision or intervention (Figure S11). To complete the reaction preparation, 0.5 mL of reaction solvent was added by hand with a six-channel volumetric pipet to the solids-containing wells, and the plates were stacked and sealed. Although automated liquid dispensing could have been used for this step, manual loading of solvents was preferred for its simplicity.^74^

With a viable approach to efficiently load and operate RCF reactions at high temperatures developed, we next investigated the preparation of the reaction product for analysis. Similar to the 75 mL reaction procedure, the catalyst and pulp must first be separated from the RCF liquor. To do this, the reaction mixture was transferred to a 24-well filter plate (0.2 μm) installed on a vacuum manifold using wide-bore pipet tips to increase the transfer of solids (Figure 1B, step 5; see Table S1 for equipment details).^57^ To increase total material recovery, the wells were washed three times with 0.5 mL of a wash solvent (*vide infra*) to ensure maximal transfer of the reaction mixture followed by a final 1 mL wash of the filter.

To quantify the delignification and monomer yield, the combined reaction mixture and wash solvent must be brought to a fixed volume of deuterated solvent with an internal standard at a known concentration. The combined reaction mixture and washes were evaporated under flowing air for ∼20 min until dry. The dried oil was then redissolved in 1 mL acetone-*d*_6_ containing 1 g/L 1,3,5-tri-*tert*-butylbenzene (TTB) as an internal standard for both GC-FID and ^1^H NMR. Half of this solution (0.5 mL) was added to an NMR tube and capped, whereas the remaining solution was analyzed via GC-FID for aromatic monomer content.

### Reproducibility of the HTP-RCF Method

Achieving a high degree of reproducibility is critical for the HTP-RCF method to be useful. This is made difficult by the fact that RCF reactions consist of liquids, gases, and multiple solids, which are each subject to distinct heat and mass transfer limitations.^4^ In the HTP-RCF method proposed here, the stacked-plate configuration introduces heat transfer differences across the plates where reactions occurring at the outer wells of the plate are potentially heated more quickly than the internal wells. Furthermore, at the reduced scale of HTP-RCF, small material losses can influence the results.^57^ Given these challenges, we sought to explore the reproducibility of the method. In conventional-scale RCF reactions (e.g., a 75 mL Parr reactor), full recovery of the extracted lignin is possible through washing of the residual solid pulp and quantitative transfers at each step. For HTP-RCF, complete recovery of the reaction product was not tractable despite numerous washings of the well and filters. To account for this transfer loss from the reaction wells to analysis in the initial HTP-RCF reactions described above (Figures 3 and 4B), a surrogate was added to each well after the reaction before filtration (TTB or dimethoxybenzene (DMB)), and concentration measurements were scaled by eq 1.
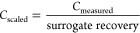
1

For eq 1 to accurately account for transfer losses, the surrogate must be transferred in proportion with the desired analytes. In the preliminary investigations using TTB or DMB as the surrogate, the scaling relationship in eq 1 was an assumption based on the structural similarity between the lignin components and the surrogate and only validated by the good agreement between HTP-RCF reactions and reactions at the 75 mL scale (Figure 4B). We posited that further efficiency gains could be achieved by including the surrogate in the initial reaction solvent, which would prevent the need to add a known amount of surrogate to each well individually after the reaction.

A surrogate is necessarily inert, nonvolatile, soluble in the reaction system solvent(s) and can be measured by the analytical method used, which here was GC-FID. Adding the surrogate to the solvent prereaction imposes the additional constraint that it must be inert in RCF conditions. This presents a trade-off where molecules that are structurally similar and could be assumed to have recoveries most similar to RCF oil likely also contain functional groups that could be reactive under RCF conditions. Octadecane was chosen for investigation as a surrogate for its inertness; however, given the structural differences between octadecane and the aromatic molecules in RCF oil, it was necessary to validate its use.

**Figure 6:**
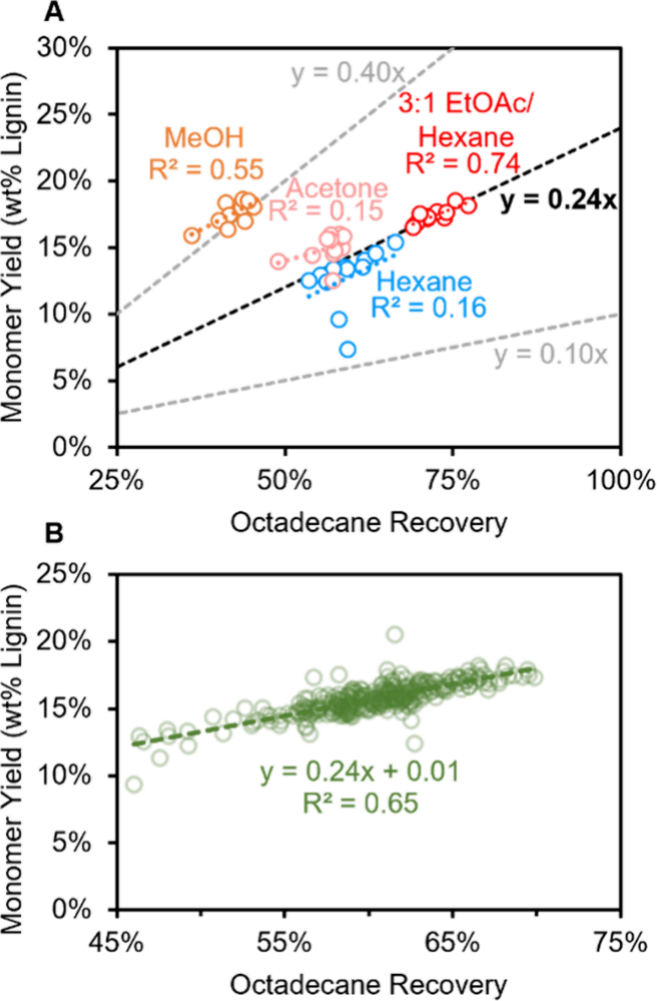
Validation of octadecane surrogate. (A) Results of wash solvent screening showing a positive correlation between octadecane recovery and unscaled monomer yield. The black dashed line (*y* = 0.24*x*), included for reference, is the scaling that recovers identical monomer yields to a 75 mL batch reaction. (B) Correlation of octadecane recovery and unscaled monomer yield for 295 replicate samples conducted across 10 HTP-RCF reactions. Conditions: 50 mg poplar, 15 mg Ru/C, 200 °C, 6 h, 0.5 mL 1:1 IPA/MeOH with 2 mg/mL octadecane.

An HTP-RCF reaction was performed with 2 mg/mL octadecane surrogate dissolved in the reaction solvent, 1:1 IPA/MeOH (0.5 mL solvent, 50 mg poplar, 15 mg Ru/C, 200 °C, 6 h). The wash solvents ethanol, methanol, ethyl acetate, acetone, and isopropanol and their 25, 50, and 75% mixtures with hexane (21 solvent combinations, including hexane) were screened for their ability to effectively transfer the postreaction RCF product, including the octadecane surrogate, to filtration and downstream analysis (step 5 in Figure 1B). Mixtures were evaluated for the accuracy and precision to which their yields reflected results from 75 mL batch reactions (Figure 4B) across 12 replicate samples. The wash solvent composition had an appreciable effect on the recovery of the reaction mixture, with measured unscaled monomer yields ranging from 12.5 ± 0.8% (3:1 IPA/hexane) to 18.1 ± 0.8% (EtOAc). Figure 6A shows the unscaled monomer yield as a function of the octadecane recovery for a select set of solvent mixtures. A positive correlation between the unscaled RCF monomer yield and octadecane recovery was observed for most solvents, but the proportionality and therefore the resulting scaled monomer yields varied widely. The black dashed line in Figure 6A represents the relationship between octadecane and RCF monomer recovery that results in a scaled yield equal to that from the 75 mL batch reaction (24 ± 1%). Polar protic solvents such as MeOH preferentially recovered RCF oil, giving scaled yields that were much higher (41 ± 2%) than those obtained in 75 mL batch reactions. The mixture of 3:1 ethyl acetate/hexane demonstrated a consistent scaled monomer yield of 24.1 ± 0.4% (Figures S12 and S13). Importantly, scaling by octadecane recovery decreased variability in the monomer yields across the 12 replicates using the 3:1 ethyl acetate/hexane solvent as measured by the coefficient of variation (CV = standard deviation of replicates/average of replicates; unscaled yield: 17.4 ± 0.6, CV = 3.4%; scaled yield: 24.1 ± 0.4%, CV = 1.84%). A reduction in CV was observed for 15 of the 21 solvent combinations tested, albeit to varying degrees (Table S9). Together, these results indicate that use of octadecane as a surrogate in conjunction with 3:1 EtOAc/hexane as a wash solvent is a viable way to decrease variability in measurements across replicate samples.

We next sought to further confirm the use of the octadecane surrogate and investigate potential sources of systematic errors arising from the plate, position on the plate, and batch-to-batch variance. During an experimental campaign run over 12 weeks, 10 separate HTP-RCF experiments were performed. Each experiment consisted of 10 reaction plates (10 plates × 24 samples per plate = 240 total samples in each experiment). As a measure of the reproducibility in the method, three replicate control reactions using the same poplar as above were run on each plate in wells A1 (corner position), B3 (internal position: second row, third column), and C5 (internal position: third row, fifth column) for a total of 30 unique sample positions in a single experiment (Figure S1E).

**Figure 7:**
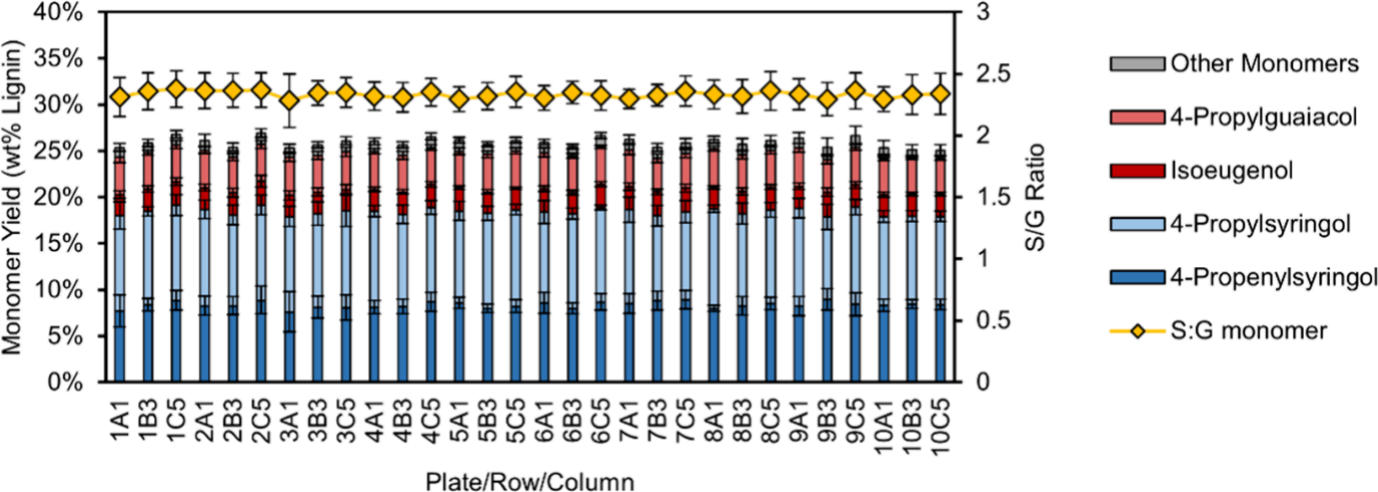
Reproducibility of the HTP-RCF protocol. Average of monomer yield and S/G ratio across 10 reactions for each well position in an experiment of 10 plates. The number label corresponds to the plate number (1–10), the row (A–D), and the column number (1–6) to give each well a unique identifier. Conditions: 50 mg poplar, 15 mg Ru/C, 200 °C, 6 h, 0.5 mL 1:1 IPA/MeOH with 2 mg/mL octadecane. These experiments were conducted over the course of a 12 week experimental campaign.

Across these 300 replicate measurements (3 controls per plate × 10 plates per experiment = 30 control reactions per experiment, leading to 300 total replicates across the 10 experiments), only five failed reactions occurred, resulting from one entirely leaked plate with no samples recovered (three control samples lost), one single dry well indicating an isolated leakage, and one overdosed poplar solids (83 mg poplar when 50 mg was desired). A similar relationship between octadecane recovery and unscaled monomer yield was observed, confirming the combination octadecane surrogate and 3:1 EtOAc/H_2_O wash solvent as a viable scheme for product recovery (Figure 6B). The average scaled monomer yield was 26 ± 1%, and specific averages for the 30 unique well positions ranged from 25.3 ± 0.7 (10C5) to 27 ± 2% (2C5). The three positions on plate 10, the topmost plate, exhibited the three lowest average monomer yields, but no clear trends with either plate number or well position were observed outside of this (Figure 7). *t* tests for statistical significance were performed across 435 unique interactions between the wells (two-sided, unequal variance). A total of 87 of the well comparison tests were significantly different (α = 0.05), which indicate some systematic bias based on well position. Nonetheless, these variations across wells were small (<2% absolute yield differences), and the method should thus be able to provide adequate experimental resolution. Minimal variation in monomer yield from batch to batch was measured; however, a small but significant increase in the S/G ratio was observed, likely due to variation in the GC-FID quantifications (Figure S14).

### Application to Switchgrass Population

A recent technoeconomic analysis of an RCF biorefinery demonstrated the critical importance of achieving high delignification extent and monomer yield.^31^ Given the substantial influence that substrate choice has on monomer and oil yield,^25−28^ the economic outlook of an RCF biorefinery is expected to be sensitive to the feedstock. However, RCF variability up to this point has mainly focused on biomass type (hardwood versus softwood) or genus (i.e., poplar versus birch) rather than intragenus or intraspecies variability. Happs et al. recently established that variations in biomass yield (dry metric ton per hectare) and composition (mass fraction of fermentable sugars) within undomesticated poplar^53^ and switchgrass^75^ populations were key drivers of economic and sustainability metrics for ethanol biorefineries. Biomass yield was predicted to be the primary driver of ethanol price, but composition would provide an edge when similar yielding genotypes were considered. A key aspect of these studies was the use of HTP analysis to ascertain the degree of variability at the population level. Lignin extraction and monomer yield may also show similar variation within a species with potentially large economic consequences; however, the low throughput of conventional RCF reactions has proven to be a barrier to exploring this variance.^40,41^

**Figure 8:**
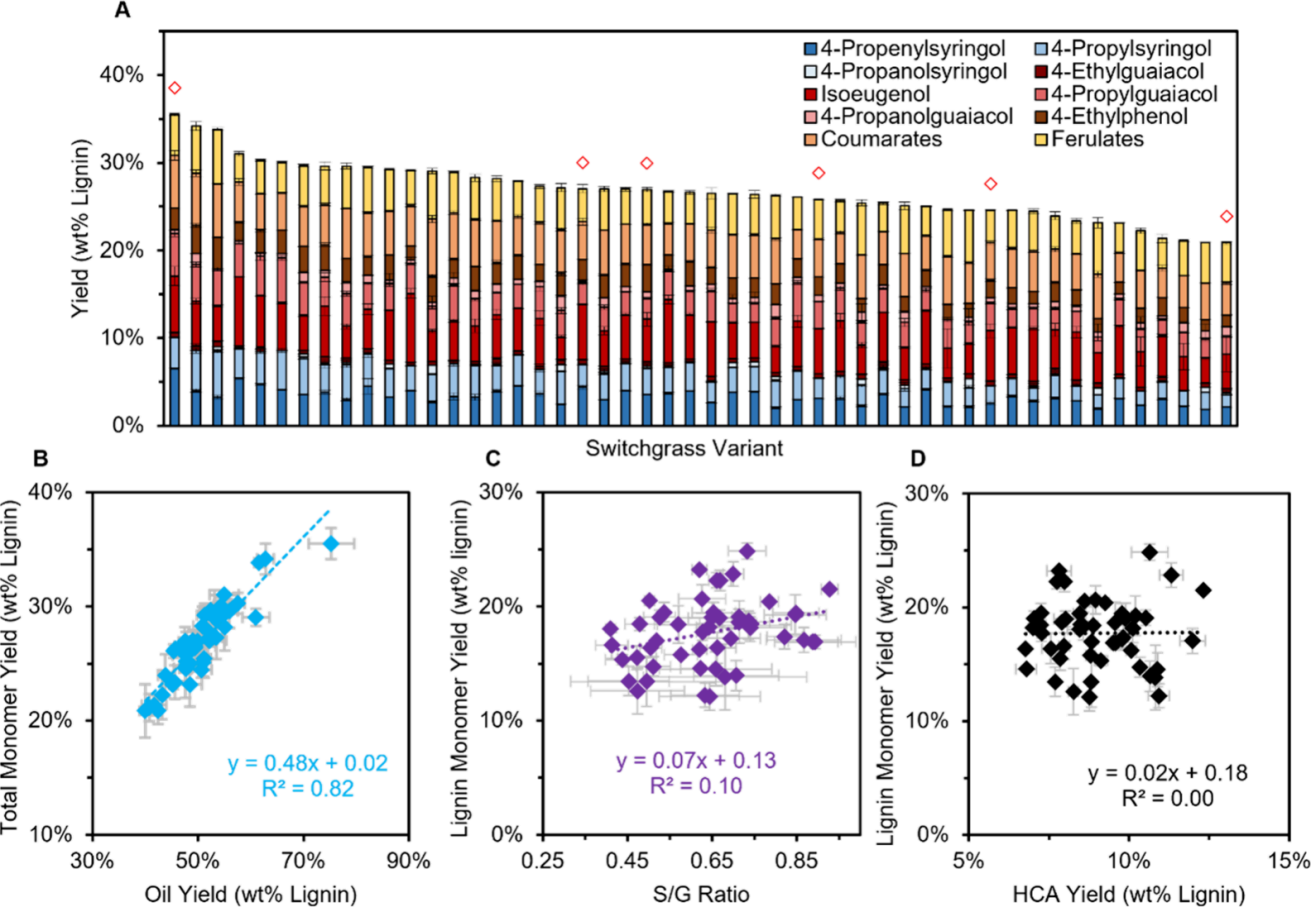
Screening 50 switchgrass samples using the HTP-RCF method. (A) Monomer yields with multiple products deriving from hydroxycinnamate units grouped into ferulates and coumarates. Red diamonds indicate samples selected for validation in 75 mL batch reactors. Relation between (B) oil yield and total monomer yield, (C) S/G ratio and lignin monomer yield (excluding hydroxycinnamate-derived products), and (D) total hydroxycinnamate yield (ferulates + coumarates) and the lignin monomer yield. Conditions: 50 mg switchgrass, 15 mg Ru/C, 0.5 mL 1:1 IPA/MeOH, 6 h, via workup “Method II”. Error bars are the standard deviation of triplicate measurements. Table S10 provides the quantitative data in this figure.

To demonstrate the utility of the method, we screened the RCF performance of 50 unique switchgrass samples in triplicate (44 unique genotypes, 6 repeated genotypes in alternative growth conditions) in the HTP-RCF system (Figure 8, Table S10). To allow for yields to be substrate-dependent (extraction-limited), a catalyst loading of 30 wt % was selected (15 mg Ru/C, 50 mg switchgrass). Conventional RCF monomers such as 4-propyl, propenyl, ethyl, and propanol substituted monomers were quantified via GC-FID, and hydroxycinnamate products (derived from *p*-coumaric and ferulic acid) were identified and quantified using the ^1^H NMR method (Figure S4). To calculate the oil yield, the contributions of the hydroxycinnamate products were subtracted from their respective S and G regions, and their yields were added to the total oil yield using the known molecular weight rather than the calibration mass described above (Supporting Information S1.6).

Lignin-derived (nonhydroxycinnamate) aromatic monomer yields ranged from 12 to 25% (average of 50 samples, *x̅* = 17.7%, standard deviation of 50 samples, σ = 2.6%) with high selectivity to propyl and propenyl side chains as observed for the exploratory reactions on poplar. Hydroxycinnamates contributed an additional 7–12% to the total monomer yield (*x̅* = 9.9%, σ = 1.4%), leading to total monomer yields in the range of 21–36% (*x̅* = 26.7%, σ = 3.2%). The average standard deviation of triplicate measurements for total monomer yield was 1.2%, indicating high reproducibility across reaction wells. Free carboxylic acids were not observed in the ^1^H NMR spectrum for coumarate- and ferulate-derived products, indicating complete esterification.^26^ The side chain double bonds in the hydroxycinnamate-derived products were partially hydrogenated, leading to a mixture of saturated and unsaturated products, similar to lignin-derived monomers. Furthermore, 4-ethylphenol and 4-ethylguaiacol were observed, indicating partial decarboxylation of the hydroxycinnamates (Figure 8A; 4-ethylsyringol was not detected). A positive relationship between oil yield and total monomer yield was observed (*R*^2^ = 0.82), and the ratio of monomer yield to total oil yield was consistent at ∼0.48 (Figure 8B). Only a weak correlation between the S/G ratio and lignin monomer yields (*R*^2^ = 0.10, Figure 8C) was measured, indicating that the S/G ratio does not dictate the RCF monomer yield, in line with previous results on naturally variant poplar.^41^ Lignin monomer yield and total hydroxycinnamate yield showed no correlation (Figure 8D), but there was a positive correlation between *p*-coumarate and ferulate-derived products (Figure S15). In the context of a switchgrass-based biorefinery, these results demonstrate that the economic outlook (minimum selling price of the RCF oil) could be substantially affected by substrate choice alone, with higher yielding variants leading to more favorable economics.

**Figure 9:**
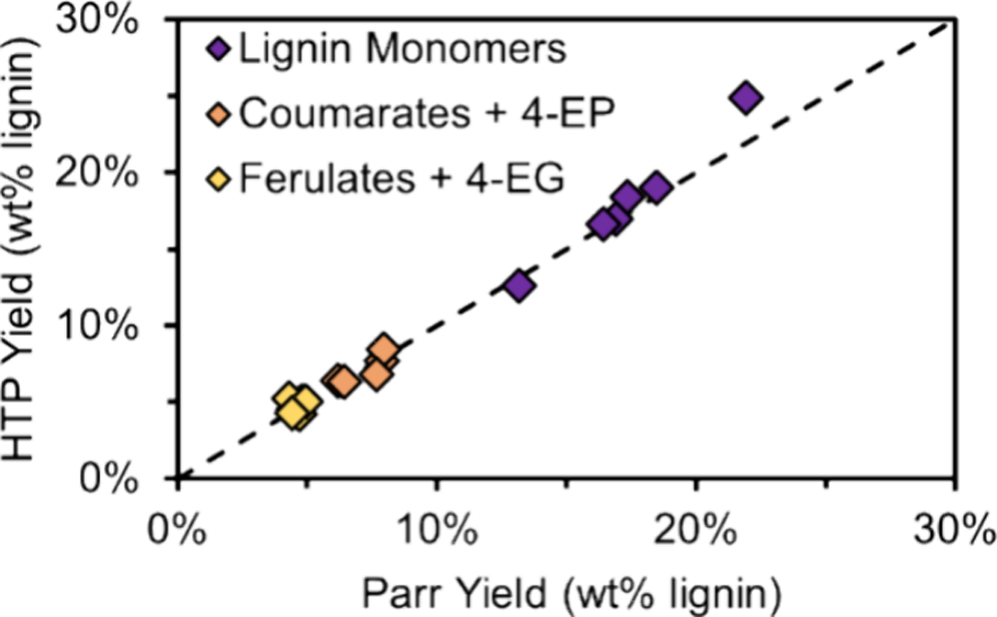
HTP conditions: 50 mg switchgrass, 15 mg Ru/C, 0.5 mL 1:1 IPA/MeOH, 200 °C, 6 h, triplicate reactions, workup “Method II”. Seventy-five milliliter conditions: 2 g switchgrass, 400 mg Ru/C, 30 mL MeOH, 225 °C, 3 h, duplicate reactions. Table S11 provides the quantitative data in this figure.

Given the significant differences between conventional and HTP-RCF scales and procedures, we last sought to validate the HTP method by comparing HTP reaction results to RCF conducted in 75 mL batch reactors. Although the conditions chosen for HTP-RCF reactions were replicable at the 75 mL reactor scale, as described above, conventional batch reactions are typically run at higher temperatures (225–250 °C) to achieve higher extents of lignin extraction. We thus aimed to demonstrate that the substrate variability measured with the HTP system was not due to the choice of solvent or reaction conditions and that the less severe HTP-RCF conditions chosen can capture trends in substrate behavior at our standard 75 mL reactor conditions. We selected six switchgrass samples, including the variants that gave maximum and minimum monomer yield, for validation in the 75 mL reactors. Reactions were conducted in duplicate in 75 mL reactors at 225 °C for 3 h with 30 bar H_2_ using 20 wt % loading of 5 wt % Ru/C as the catalyst to ensure full conversion of the extracted lignin to monomers. Nearly identical values of both lignin monomer yield and yields of hydroxycinnamate-derived products were observed in 75 mL reactions and in the HTP-RCF system (Figure 9, Table S11). This indicates that the lower temperature (200 °C) in the HTP-RCF was sufficient to extract lignin to a similar extent as the 75 mL scale reactions conducted at 225 °C.

## Discussion and Conclusions

In this work, we developed a small-scale HTP-RCF reactor system capable of running 240 RCF reactions using 50 mg of biomass and 0.5 mL of solvent in each reaction. Reaction parameters such as catalyst loading and reaction solvent were investigated, and the HTP reaction results closely matched results using 75 mL batch reactors. Several aspects of the pre- and postreaction protocol were adapted to increase throughput, including the development of a ^1^H NMR spectroscopy method to quantify extracted lignin in lieu of gravimetric oil mass. The NMR method presented here has several advantages that make it convenient for use in the HTP-RCF setup, as well as a viable alternative to gravimetric measurement in other contexts. It requires only a small amount of oil and avoids the need for rotary evaporation and liquid–liquid extraction. Furthermore, the method relies on aromatic signals from a ^1^H NMR spectrum and is thus unaffected by the presence of nonlignin organic soluble components that would typically inflate the gravimetric mass provided that these components do not exhibit resonances in the integration region. This method can also quantify monomers in RCF oils for reactions with known selectivity. HTP-RCF was performed on 50 switchgrass variants using the HTP method, revealing a wide range of monomer yields that could have important consequences for the biorefinery. Monomer yields showed no correlation with the S/G ratio, similar to our previous work on five genotypes of poplar.^41^ Instead, a positive correlation between extracted lignin and monomer yield was observed, indicating that improving RCF monomer yields may rely on increasing lignin extractability. The method was also validated by repeating reactions in 75 mL batch reactors for a subset of switchgrass variants at our standard RCF reaction conditions for this scale, and nearly identical yields were obtained.

If the switchgrass reactions shown in this work had been performed solely in 75 mL batch reactors, the process would have required 150 independent reactions consuming 300 g of biomass (2 g per reactor), 4.5 L of MeOH (30 mL per reactor), and 60 g of Ru/C (400 mg per reactor). Assuming that six reactors could be successfully run per day, this amounts to 25 days of reactor use and workup. In contrast, the HTP-RCF system only required 7.5 g of biomass, 75 mL of solvent, and 2.25 g of Ru/C. Low material loadings preserved valuable biomass substrates and limited excess solvent use. Minimizing required catalyst loading will be critical for investigations where catalysts need to be synthesized prior to use rather than purchased commercially. The 150 reactions required less than a full 10-plate experiment (240 reactions), and the reaction and analysis (GC-FID + ^1^H NMR) were completed within 7 days, clearly demonstrating the time and material advantage of the HTP-RCF system. In total, we estimate a 10–15× increase in throughput per unit of researcher time (Table S12).

Notably, the described reaction system is limited by key factors that prevent its ability to fully represent conventional RCF reactions. Although the HTP-RCF results closely match results obtained using 75 mL batch reactor experiments, we stress that the reaction conditions used here should serve as an example of the utility of the system and a means to guide future experimentation. The number of interrelated variables is too great to guarantee that all relevant phenomena are adequately represented by the parameter set chosen in this work, such as solvent and catalyst loading. Although H_2_-free RCF schemes are becoming increasingly popular, the majority of RCF reactions still utilize external H_2_ gas, and catalyst performance appears to be sensitive to this.^7,17,19,68^ As RCF schemes evolve to utilize different process configurations, the lack of stirring may not capture relevant mass transfer effects. Reactions using the 1:1 IPA/MeOH system did not achieve full lignin extraction extents and thus may represent mixed effects from variation in extractability and linkage abundance across substrates. Additionally, conversion of bio-oils to fuels requires further deoxygenation at higher temperatures and pressures, which are not accessible in the current design.

The inclusion of NMR as an analytical method requires the removal of the reaction and workup solvents, and therefore, direct application of the protocol would be unfeasible for higher boiling solvents such as ethylene glycol without additional method development such as a liquid–liquid extraction with an immiscible solvent.^47^ Nonetheless, the method may find application for the expedited screening of catalysts and solvent systems. The system is particularly well suited for the screening of natural variant populations to enable genome-wide association studies that require large numbers of unique and therefore valuable biomass samples. Additionally, use of the plate reactors can likely be extended to other high-pressure and high-temperature applications aside from RCF.^49,76^

## Methods

The Supporting Information contains a full description of the materials and methods used in this paper including reaction setup, analysis with GC-FID and the ^1^H NMR method, equipment list, and supplementary figures and tables. All monomer and RCF oil yields were calculated relative to the amount of lignin in the initial biomass loaded into the reactor.

### HTP-RCF

Biomass and Ru/C were loaded into wells using a solids-loading robot, and 0.5 mL of the reaction solvent was added by hand with a volumetric pipet. After sealing, the plates were heated either in a static oven or by 200 psi steam for the desired reaction time. After the reaction, the reaction mixture was filtered, and the solvent was evaporated under flowing air. The RCF oil was redissolved in acetone-*d*_6_ and analyzed via GC-FID (aromatic monomer yield) and ^1^H NMR (RCF oil yield).

### RCF in 75 mL Batch Reactors

Biomass and Ru/C were added to the bottom of a 75 mL Parr reactor followed by the desired amount of solvent. The reactor was sealed and pressure tested with helium, and when desired, H_2_ was added to the reactor at a pressure of 30 bar. For reactions mimicking HTP-RCF conditions, a valve was opened to allow the reactor headspace to equilibrate with air, and then helium was added to a pressure of 5 bar to dilute the air. The reactors were heated for the desired amount of time, and then cooled in an ice bath to halt the reaction. The reaction solvent was evaporated in a rotary evaporator, and liquid–liquid extraction was performed with ethyl acetate and water. The combined organic fractions were evaporated in the rotary evaporator, and the mass of the isolated lignin oil was measured. The oil was dissolved in acetone, and aromatic monomers were measured with GC-FID.

## Supporting Information Available

Full description of the materials and methods used in this paper including reaction setup, analysis with GC-FID and the ^1^H NMR method, equipment list, and supplementary figures and tables (PDF)
Plate design drawings (ZIP)
