# Supplementary material for: Design and Validation of a High-Throughput Reductive Catalytic Fractionation Method
Source: JACS Au. 2024 Jun 5;4(6):2173–87. doi: 10.1021/jacsau.4c00126 (PMC11200236; doi:10.1021/jacsau.4c00126)
Supplement: Supplementary file 1 — au4c00126_si_001.pdf [file au4c00126_si_001.pdf]

Supporting Information

Design and validation of high-throughput reductive catalytic fractionation technique

Jacob K. Kenny,[1,2,3,‡] Sasha R. Neefe,[1,3,‡] David G. Brandner,[1,3,‡] Michael L. Stone,[1,3,‡] Renee M. Happs,[1,3] Ivan Kumaniaev,[4,5] William P. Mounfield III,[4] Anne E. Harman-Ware,[1,3] Katrien M. Devos,[3,6,7,8] Thomas H. Pendergast IV,[3,6,7,8] J. Will Medlin,[2] Yuriy Román-Leshkov,[4,*] Gregg T. Beckham[1,2,3,*]

1. Renewable Resources and Enabling Sciences Center, National Renewable Energy Laboratory, Golden CO 80401, USA
2. Department of Chemical and Biological Engineering, University of Colorado, Boulder, CO 80303, USA
3. Center for Bioenergy Innovation, Oak Ridge, TN, 37830, USA
4. Department of Chemical Engineering, Massachusetts Institute of Technology, Cambridge, MA 02139, USA
5. Department of Organic Chemistry, Stockholm University, SE-106 91 Stockholm, Sweden
6. Institute of Plant Breeding, Genetics and Genomics, University of Georgia, Athens, GA, 30602, USA
7. Department of Crop and Soil Sciences, University of Georgia, Athens, GA, 30602, USA
8. Department of Plant Biology, University of Georgia, Athens, GA, 30602, USA.

[‡] Denotes equal contribution
* Correspondence: yroman@mit.edu; gregg.beckham@nrel.gov

1. Supplemental methods

1.1 75 mL scale RCF reactions

RCF reactions were conducted in 75 mL batch autoclave reactors (Parr® Instruments, Moline, IL). A magnetic stir bar was placed in the bottom of the reactor and 2 grams of substrate and 400 mg of Ru/C (the catalyst is 5 wt% Ru loading) was added. The catalyst was then wetted with 400 mg of deionized water to prevent ignition of the solvent. Methanol was then added to bring the total volume to 30 mL (mass measured with a balance), and the reactor was sealed, and pressure tested at 90 bar. The reactor was purged three times with He, and then filled with H_2. The reactor was heated to 225°C in approximately 30 minutes and held for three hours. After reaction, the reactors were cooled in ice water to room temperature. Approximately 10 mL was filtered through a 0.22-micron syringe filter. To measure the oil yield, a 0.5 mL aliquot was taken and dried under flowing air and redissolved in a known volume of 1 g/L 1,3,5-tri-tert-butylbenzene (TTB) in acetone-d_6. A ^{1}H NMR experiment was performed on a Bruker 400 or 600 MHz instrument with 32 scans and a delay of 3 seconds. A sample was prepared for GC-FID analysis by combining 0.5 mL of the RCF liquor with 0.5 mL of a 2 g/L solution of TTB in methanol.

1.2 Liquid-liquid extraction for isolation of lignin oil from 75 mL batch reactions

To calibrate the ^{1}H NMR method for measuring the NMR oil yield, the lignin fraction was isolated from the RCF reaction mixture via liquid-liquid extraction. Approximately 5 mL of the reaction mixture from the 75 mL RCF batch reactions was added to a 100 mL round bottom flash. The methanol was evaporated in a rotary evaporator, and the resulting oil was subjected to liquid-liquid extraction with ethyl acetate and water to isolate the lignin oil from water soluble components. The water layer was washed two additional times with ethyl acetate. The three organic phases were combined and evaporated in a rotary evaporator, yielding the lignin oil. The lignin oil was then redissolved at a known concentration in acetone-d_6 with TTB as an internal standard. Aliquots of this stock solution were combined with additional acetone-d_6 with TTB (1 g/L) to give solutions of various concentrations. A ^{1}H NMR experiment was performed using 32 scans and a 3 second delay (d1) on a Bruker 400 MHz instrument equipped with Prodigy cryoprobe. The S (6.2-6.6 ppm) and G (6.6-7.2 ppm) regions were integrated relative to the TTB peak (~7.3 ppm), giving the moles of the respective units after adjusting for the number of hydrogen atoms.

1.3 Procedure for initial reaction testing in HTP plate reactor (Method I)

Initial reactions conducted in the plate reactors were analyzed using a slightly modified procedure compared to the optimal throughput presented in the main text **Figure 1** and described in section **S1.4**. After affixing O-rings to the bottom of the required plates, poplar and Ru/C were massed and added to the wells by hand. The solvent was then added with a volumetric pipette, and the plates were stacked and sealed. The sealed plate reactor was placed in a static oven set to the desired reaction temperature, marking the beginning of the reaction time. To account for transfer losses during the workup, a surrogate was added to each well post reaction. Identical results were obtained from the screening of two internal standards: 1,2-dimethoxybenzene (DMB) and 1,3,5-tri-tert-butylbenzene (TTB). To increase the material for recovery, three identical reactions wells were combined prior to performing the workup procedure. The combined wells were first filtered through filter paper. The wells were washed with 3 x 0.5 mL of isopropanol, and the filter was washed one additional time with 1 mL isopropanol. Reaction and wash solvent were evaporated in a rotary evaporator. The resulting RCF oil was analyzed via gas chromatography with flame ionization detector (GC-FID), and the total amount of extracted lignin (referred to as the RCF oil yield) was measured both gravimetrically and via ^{1}H NMR. Data presented is the average of at least two replicates. Data shown in **Figure 3** were acquired using this procedure.

1.4 HTP-RCF procedure (Method II)

To enable the processing of samples with higher throughput, modifications were needed to the initial Method I. Below is a list of the key modifications to Method I.

1. Use of a solids loading robot (Symex Powdernium) to dispense biomass and catalyst into plate wells.

2. Loading of the plates into a larger pressure vessel capable of dosing high pressure steam instead of loading into an oven for heating reaction plates.

    a. Batch reactors are typically heated by commercially available internal resistance heaters with a thermocouple to read the internal temperature of the fluid. Given the custom design of the plates, we instead utilized a 2-gallon Parr® reactor capable of constantly flowing saturated steam. The steam pressure, and thus the temperature was controlled by a control valve upstream. The sealed plates were loaded into the steam reactor without preheating, and a thermocouple was placed in contact with the outside of the plate reactors to measure the steam temperature (the reactor temperature was not controlled by this thermocouple). After the steam reactor was sealed, saturated steam at 200 psig (approximately 198 °C), was introduced into the reactor, marking the beginning of the reaction. After the desired reaction time (6.5 hours after introducing steam), steam flow was stopped, and cooling water was introduced into the reactor which immediately began cooling the reactor. The reactor was filled with cooling water and then emptied to cool the reactor, and this cycle was repeated until the internal thermocouple read 35 °C. The plate reactor was removed from the steam reactor. The

cooling water also served as a safety mechanism, which could be used to quench the reaction in the case of malfunction or leaking.

3. Use of 24-well filter plates instead of individual filtrations with filter papers.

4. Evaporation of the solvent in an air dryer capable of drying up to 50 samples at a time instead of a rotary evaporator.

To accommodate the large number of samples, the HTP-RCF procedure was modified, and setup, operation, and workup were conducted over multiple days. On the first day, O-rings were placed on the bottom of eleven 24-well reactor plate (ten for the reaction, and one additional plate to seal the top plate) which served to seal the plate below. A solids handling robot was utilized for the loading of biomass and catalyst into the wells. Solids dispensing hoppers were loaded with approximately 250 mg of biomass. A solution of 2 g/L octadecane (C18, surrogate standard) in the desired reaction solvent (1:1 v/v IPA:MeOH for reactions other than where reaction solvent is noted) was prepared by massing each component.

The following day after solids are loaded into the wells, 0.5 mL of the reaction solvent was added to each well using a 1000 µL positive displacement pipet. Plates are labeled with rows A (furthest from user) to D (closest to user) and columns 1 to 6 (left to right), stacked, and numbered from 1 (bottom) to 10 (top). The 11th empty plate was stacked to seal plate 10. Plates were then mounted onto the bottom holder plate, and the top holder plate was installed. Twelve 14-inches all-threads were prepared by adding ten spring discs/Belville washers to each along with three nuts. All threads were then routed through the top plate and screwed into the bottom plate. The nuts were then tightened in a star pattern to compress the spring discs and seal the O-rings. To ensure proper sealing, unrestricted travel of the nuts through the tightening length of the all-thread is necessary to allow for perceived torque to be an indication of tightness of the compression discs. The reactor was then inserted into a larger batch reactor capable of dosing high pressure (200 psig) steam (referred to here as the "steam reactor"), which was then sealed. Saturated steam at 200 psig was introduced to the steam reactor to heat the plates to 196-198 °C (temperature is monitored using a thermocouple in contact with the outside of the plates), marking the start of the reaction. At the desired reaction time (usually 6.5 hours after the introduction of steam in this work) the steam was shut off and cooling water was introduced to the steam reactor. Cooling water was drained and then added again five times or until the thermocouple read 35 °C, at which point the reactor was opened, and plates were lifted out. Residual water was blown off the plates with compressed air and left to continue cooling and drying overnight.

On the third day, a 3:1 v/v Ethyl Acetate to Hexane solution ("workup solvent") was prepared using graduated cylinders. A vacuum manifold was loaded with 24, 4-mL vials labeled A1-D6 and a 24-well 0.2µm Thomson Rapid Clear filter plate from Thomson Instrument Company was placed on top of the manifold. The top sealing plate was removed, revealing the first plate. Plates were worked up one at a time to minimize the evaporation of the reaction solvent. Using a 6-channel 1000 µL Rainin Pipet and clean Rainin wide-orifice 1000 µL Rainin pipet tips for each well, RCF liquid product (about 0.1 mL) was transferred to the corresponding wells in the filter plate system. Using clean, regular 1000 µL Rainin pipet tips, 0.5 mL of the ethyl acetate-hexane solution was added to row A. Using wide orifice pipet tips, liquid and some solids from row A were transferred to the corresponding filter plate wells. Thie solvent addition/transfer process was repeated two more times for a total of three solvent rinses for row A, and subsequently this procedure was repeated for each of the three rows remaining in the plate. After row D has been completed, vacuum was pulled until no liquid is present in the filter plate. 1 mL of the 3:1 ethyl acetate-hexane solution was added directly to each filter plate well and vacuum was pulled again until the filter plates were dry. The filter plate and remaining solids in the reactor plate were disposed of into reactive catalyst waste, and the 4-mL vials were transferred from the manifold to the three middle rows (8 vials per row, excluding the two edge spaces) of a custom printed vial rack within a 10-nozzle Turbovap LV drying system. Air at 9 psig was blown over the samples for 22 minutes or until all solvent had evaporated from the vials. Attention was paid to minimize excess drying time to prevent RCF monomer evaporation. After drying, 1 mL of 1 mg/mL tri-tertbutylbenzene in acetone-d_6 was added to each of the 24 samples using a 1000 µL positive displacement pipet and the samples were all capped and vortexed. Samples were then uncapped one row at a time and, using a 6-channel 1000 µL Rainin Pipet and clean 1000 µL Rainin pipet tips, 150 µL of sample was transferred into Agilent sample vials with 300 µL glass inserts. GC vials are capped with Agilent vial crimp caps. Using the same 6-channel pipet and tips, 500 µL of sample is transferred from the 4 mL vials into high throughput NMR tubes and the tubes were capped.

1.5 GC-FID analysis of monomers

The small scale of HTP-RCF necessitated modifications to the standard methods for yield measurements and calculations. GC-FID requires as little as 0.1 µL for injection of liquid samples. The only further operating requirement is sufficient sample is present in the vial to ensure proper functioning of the autosampler. The necessary adaptations for analyzing the HTP-RCF samples were 1) the use of vials with inserts to reduce the required volume of total sample (~150 µL), 2) utilizing a low thermal mass modular accelerated column heater to reduce the method time to six minutes (column: Agilent DB-5, 10 m, 0.10 mm, 0.1 µm, LTM), and 3) measurement of the C18 surrogate in the GC method to enable monomer and oil yields to be corrected by recovery.

Monomer quantification was performed by injection on an Agilent 7890B GC equipped with a low thermal mass module and flame ionization detector (FID) using an injection volume of 1 μL. The sample was injected without a split to a 250 °C inlet. The temperature ramp was programmed for a 1.1 minute hold at 40 °C followed by a 90 °C/min ramp to 260 °C with no hold time and a final ramp of 150 °C/min to 325 °C with a 1 minute hold. The total method time was less than six minutes, significantly shortening the time required to inject the full run of 240 samples. All desired products are calibrated for using authentic standards.

Monomer yields (y_i) were calculated using equation 1:

$$y_i = \frac{C_{GC-FID} * V}{\frac{C18_{GC-FID}}{0.5 * C18_i} * m_{biomass} * X_{lignin}} \quad (1)$$

where C_{GC-FID} is the concentration of the monomer measured by GC-FID, V is the volume of the sample (1 mL total sample), $C18_{GC-FID}$ is the concentration of octadecane measured from GC-FID, $C18_i$ is the initial concentration of octadecane in the reaction (target was 2 g/L in 0.5 mL reaction solvent, leading to a total of approximately 1 mg of octadecane in each well), $m_{biomass}$ is the mass of biomass dispensed into the well, and X_{lignin} is the lignin content (mass percent) of the biomass as measured by pyrolysis-molecular beam mass spectrometry.

1.6 ^{1}H-NMR for delignification

The inclusion of NMR as an analytical technique required the samples to be dissolved in deuterated solvent. After the transfer and filtration of the product, samples were dried down using an air drier (Turbovap LV). The optimal drying time for the lignin products dissolved in 3:1 v/v ethyl acetate:hexane was 22 minutes at 9 psig utilizing three of the five Turbovap channels. The dried oil was then brought up in a known volume of deuterated acetone which includes 1 g/L of TTB. For HTP-RCF reactions, NMR samples were analyzed on a Bruker Avance 600 MHz instrument using 32 scans, 3 second delay, 4 dummy scans, spectral width of 12 ppm. Integrals were normalized to the internal standard TTB. Oil yield was calculated using the region 6.2-6.6 ppm as the measure of syringyl protons, and the region 6.6-7.2 ppm as the measure of guaiacyl protons. When present, the singlet arising from 4-propenylsyringol at approximately 6.67 ppm was omitted from the guaiacyl region and added to the syringyl region. *para*-Hydroxy benzoate and methyl paraben were measured by integrating the protons at 7.9 ppm, and their masses are included in the oil yield. Where observed for high-throughput reactions, primarily *para*-hydroxybenzoic acid was measured.

Substrates such as switchgrass and corn stover contain ester-linked coumaric acid and ferulate acid units. During RCF, these ester bonds can be cleaved to yield coumaric acid and ferulic acid, which can further react by being hydrogenated and/or converted to the ester of the alcohol solvent. Furthermore, they can undergo decarboxylation to yield 4-ethylphenol and 4-ethylguaiacol. This leads to ten total products that must be quantified. 4-Ethylguaiacol and 4-ethylphenol were measured on GC-FID. The eight remaining products were quantified using ^{1}H NMR. For reactions on the 75 mL scale with H_2 pressure, only the hydrogenated methyl esters were observed. However, for the high-throughput reactions, some of the unsaturated products were also observed. No carboxylic acid products were observed, indicating full conversion to the methyl esters. For calculation of the oil yield, the known compounds (coumarates, ferulates, ethyl phenol) were subtracted out of their respective syringyl and guaiacyl regions, and the lignin oil yield was calculated from the corrected regions using the S unit mass of 200 mg/mmol and G unit mass of 170 g/mol. The mass of the coumarate and ferulate products were calculated using their respective molecular weights, and added to the total oil yield.

Only the beta protons of unsaturated coumarates and ferulates overlap with the S region, and these protons were accounted for by integrating the alpha protons on the same linkages at 7.57-7.56 ppm. The G region is overlapped by the 3,5 protons of coumaric acid/methyl coumarate, the 5,6 protons of ferulic acid/methylferulate, and all aromatic protons from dihydrocoumaric acid/methylhydrocoumarate, 4-ethylphenol, dihydroferulic acid/methylhydroferulate. The yield of the known compounds was calculated and added to the lignin oil yield obtained from the addition of the S and G peaks to give a total oil yield (equations 2-5). The regions used are listed in **Table S7**, and sample spectra are provided in figures **S2-S3**.

$$\mathbf{S} = 1.5 * M_S * n_{TTB} * \frac{I_S + I_{P=S} - I_{HC_\beta}}{I_{TTB} * R_{C18}} \quad (2)$$

$$\mathbf{G} = M_G * n_{TTB} * \frac{I_G - I_{P=S} - I_{MC} - 2I_{MHC} - 2I_{MF} - 3I_{MHF} - 2I_{EP}}{I_{TTB} * R_{C18}} \quad (3)$$

$$\mathbf{HC_i} = N * MW_{HC_i} * n_{TTB} * \frac{I_{HC_i}}{I_{TTB} * R_{C18}} \quad (4)$$

$$\textbf{Oil Yield} = \frac{\mathbf{S+G+\sum_i HC}}{m_{biomass} * X_{lignin}} \quad (5)$$

Where **S** is the mass of syringyl oil, **G** is the mass of guaiacyl oil, **HC** is a hydroxycinnamate product, I is the integral of the indicated region, P=S is 4-propenylsyringol, MC is methyl coumarate, MHC is methylhydrocoumarate, MF is methyl ferulate, MHF is methylhydroferulate, EP is 4-ethylphenol, M_S is the calibrated mass of the syringyl unit (200 g/mol), M_G is the calibrated mass of the guaiacyl unit (170 g/mol), n_{TTB} is the moles of TTB in the sample, HC_β is the beta proton of unsaturated hydroxycinnamates, R_{C18} is the recovery of octadecane measured by GC-FID, MW is the molecular weight of the desired hydroxycinnamate product, and N is an integer to adjust for the number of protons giving rise to the resonance relative to TTB (N = 1.5 for coumarate derived products; N = 3 for ferulate derived products).

1.6.1 Impact of S/G ratio determination on the calculated oil mass

The maximum error of the NMR method (after calibration of the mass values) is constrained by two extreme cases: the first is where an entirely G-type oil is quantified as fully S, and the second is where an entirely S-type oil is quantified as fully G. We can calculate the expected error from these two cases as follows.

Case 1: G-type oil is quantified as fully S.

The integral should be divided by 3, since G units have 3 protons, and multiplied by 170 g/mol as the calibrated G value. K is a constant accounting for the internal standard mass but is not important for this example.

$$\text{True mass} = \text{K} * \frac{\text{I}_\text{G}}{3} * 170\ \frac{\text{g}}{\text{mol}}$$

However, the integral is instead erroneously divided by 2 since S units have 2 protons, and multiplied by 200 g/mol as the calibrated S value.

$$\text{Measured mass} = \text{K} * \frac{\text{I}_\text{G}}{2} * 200\ \frac{\text{g}}{\text{mol}}$$

The ratio of the measured mass to the true mass, R, is:

$$\text{R} = \frac{\text{Measured mass}}{\text{True mass}} = \frac{\text{K} * \frac{\text{I}_\text{G}}{2} * 200\ \frac{\text{g}}{\text{mol}}}{\text{K} * \frac{\text{I}_\text{G}}{3} * 170\ \frac{\text{g}}{\text{mol}}} = \frac{3}{2} * \frac{200}{170} = 1.76$$

Case 2: S-type oil is quantified as fully G-type.

$$\text{True mass} = \text{K} * \frac{\text{I}_\text{S}}{2} * 200\ \frac{\text{g}}{\text{mol}}$$

$$\text{Measured mass} = \text{K} * \frac{\text{I}_\text{S}}{3} * 170\ \frac{\text{g}}{\text{mol}}$$

$$\text{R} = \frac{\text{Measured mass}}{\text{True mass}} = \frac{\text{K} * \frac{\text{I}_\text{G}}{3} * 170\ \frac{\text{g}}{\text{mol}}}{\text{K} * \frac{\text{I}_\text{G}}{2} * 200\ \frac{\text{g}}{\text{mol}}} = \frac{2}{3} * \frac{170}{200} = 0.57$$

Thus, the maximal error induced by complete misassignment of S/G ratios as a ratio of the measured value to the true value ranges between 0.57 to 1.76.

1.7 ^{1}H NMR for monomer measurement

The NMR sample preparation for the measurement of aromatic monomers in the RCF oil is the same as in section **S1.6**. NMR spectra of individual monomers were acquired for samples using concentrations of ~5 mg/mL in acetone-d_6. Monomer identification and comparison to RCF oil spectra are shown in **Figure S8**.

1.8 Switchgrass substrates

Biomass samples were obtained from tetraploid switchgrass genome-wide association study (GWAS) panels grown at three locations with 40 genotypes originating from the UGA Iron Horse Farm (Watkinsville, GA, 33.721096, -83.310268), two genotypes from the UGA Tifton Farm grown under both well-watered (UC) and drought conditions (CV) (Tifton, GA, 31.438345, -83.580185), and three genotypes from the UTK panel grown under both low nitrogen (lowN) and moderate nitrogen (modN) conditions (Knoxville, TN, 35.903094, -83.959253). Specifics on panel establishment can be found in Davison et al.[1]Aboveground biomass was harvested in the fall of 2020 after senescence. Subsamples were chipped, dried at 60 $^\circ$ C and milled using a Wiley #4 mill with a 1 mm screen.

2. Supplemental figures

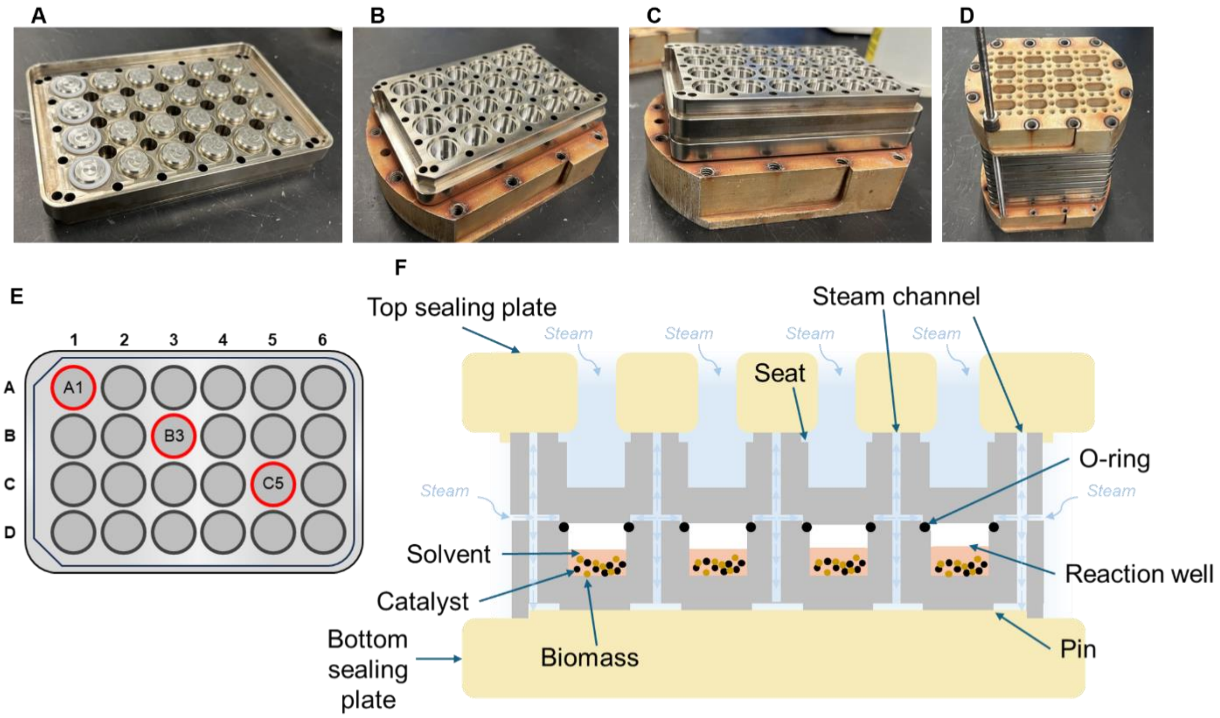

Figure S1. Images of reactor plates (**A**) underside of reactor plate with O-rings affixed to first 4 pins. (**B**) First reactor plate (without catalyst/biomass) set on top of the bottom sealing plate. (**C**) First two reactor plates stacked. (**D**) Ten reactor plates stacked with top and bottom sealing plates, with 1 of 12 disc spring laden all-threads installed. (**E**) Diagram of a plate with positions labelled, with standard control reactions marked with a red outline. Standard reactions were performed in the same well positions (A1, B3, C5) on all ten plates. (**F**) Cross section schematic showing how individual wells are sealed by pins in the plate directly above. While the PTFE O-rings used here were white in color, they are shown in black in this schematic for clarity.

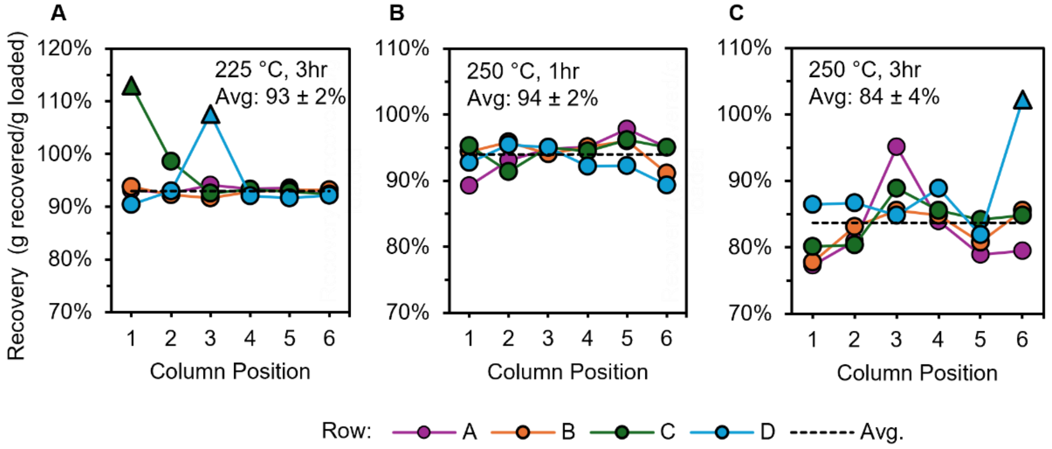

Figure S2. Reactor plate leak tests using water. (**A**) 225 °C, 1 hour. (**B**) 250 °C, 1 hour (**C**) 250 °C, 3 hours. Measurements greater than 100% (triangle marker), indicating an increase in the mass of water during the experiment, were omitted from averages and statistical analysis. Experiments were performed by pipetting 0.5 mL of water into each well in a single plate, sealing the plate with an additional plate, compressing between the endplates, and inserting into a muffle furnace set to the reaction temperature for the desired amount of time.

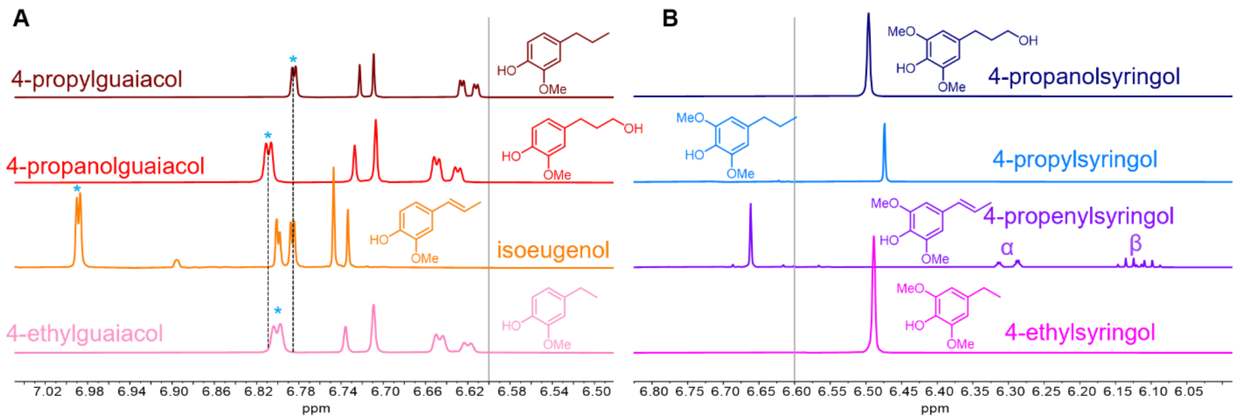

Figure S3: ¹H NMR spectra of lignin monomer model compounds. (**A**) G type monomers (**B**) S type monomers. Concentration is ~10mg/mL in acetone-d_6.

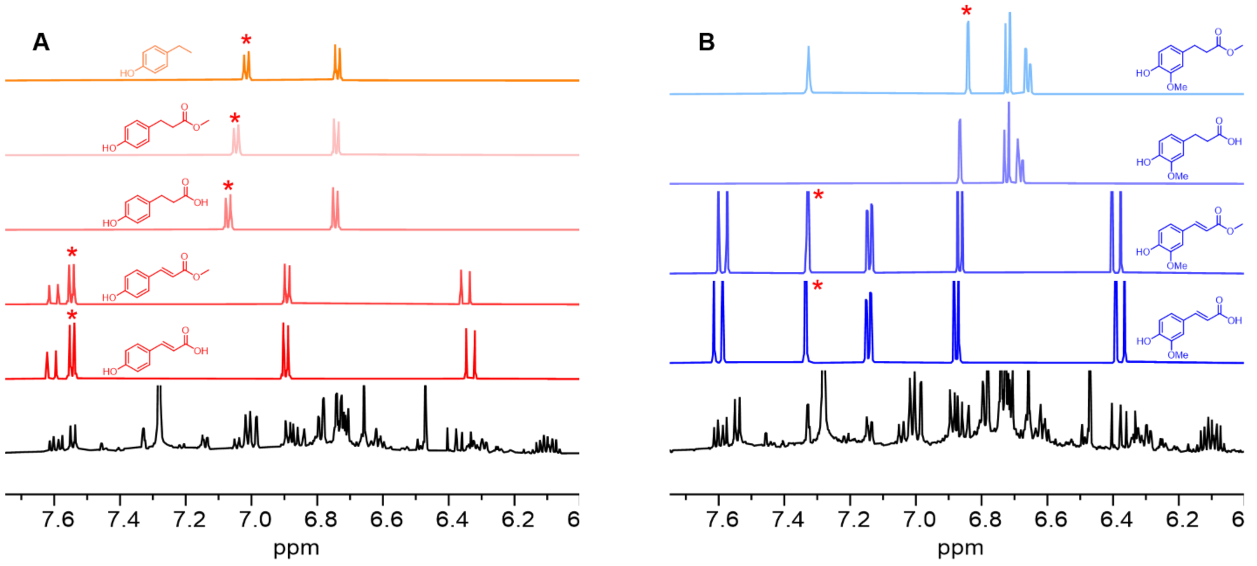

Figure S4: ¹H NMR spectra of hydroxycinnamate derived products, with resonances used for integration marked with an asterisk. (**A**) Products derived from *p*-coumaric acid. (**B**) Products derived from ferulic acid.

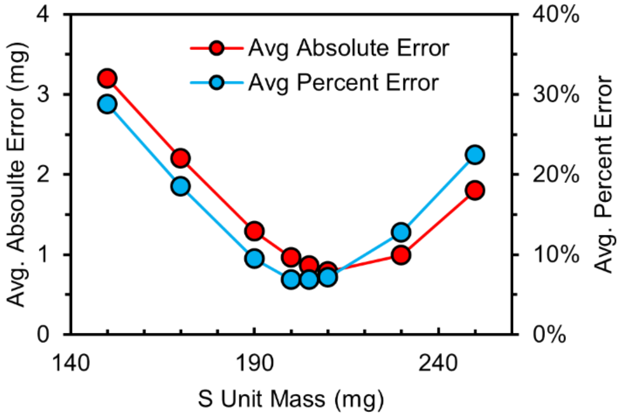

Figure S5. Example of oil yield calibration factor calculation for poplar. The percent error between the measured gravimetric mass and mass measured by NMR was minimized with an S unit value of 200 g/mol (note this is not the molecular weight but rather the average S unit plus side chain mass). G unit mass is obtained by subtracting one formyl (CH_2O) group from the S unit mass to give 170 g/mol.

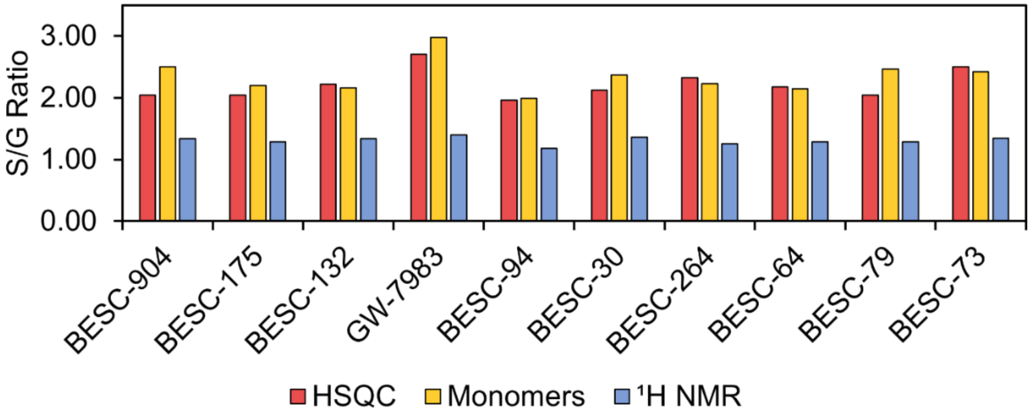

Figure S6: S/G ratio of RCF oils measured by HSQC, molar monomer yields from GC-FID, and ^{1}H NMR. Measurements are for single samples. Conditions: 2 grams poplar, 400 mg Ru/C wetted with 400 mg water, 30 mL methanol, 225 °C, 3 hours, 30 bar H_2.

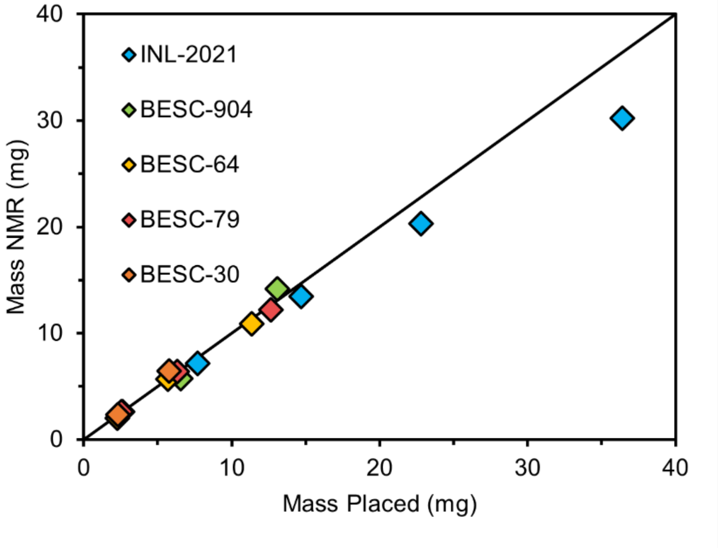

Figure S7: Gravimetric versus ^{1}H NMR oil yield for poplar RCF oils with different S/G ratios. The error between gravimetric and ^{1}H NMR calculated oil yield does not show a dependence on S/G ratio. Poplar molar S/G ratios calculated from aromatic monomers measured via GC-FID: INL-2021, 1.25; BESC-904, 2.50; BESC-64, 2.15; BESC-79, 2.47; BESC-30 2.37. Gravimetric mass is calculated from the concentration of a stock solution of oil from a particular variant. This total oil was obtained after evaporation of methanol from an aliquot of the reaction liquor (approximately 5 mL), liquid-liquid extraction in ethyl acetate and water, evaporation of the ethyl acetate, and then massing this larger quantity of oil. The oil was redissolved in a known amount of methanol to produce the stock solution. Known volumes of this stock solution were then dried under flowing N_2, and then dissolved in acetone-d_6 containing TTB (1 mg/mL) for NMR quantification of different oil concentrations. Data presented in this figure are shown as black diamonds in **Figure 5C** but are broken down in this figure by the respective genotype.

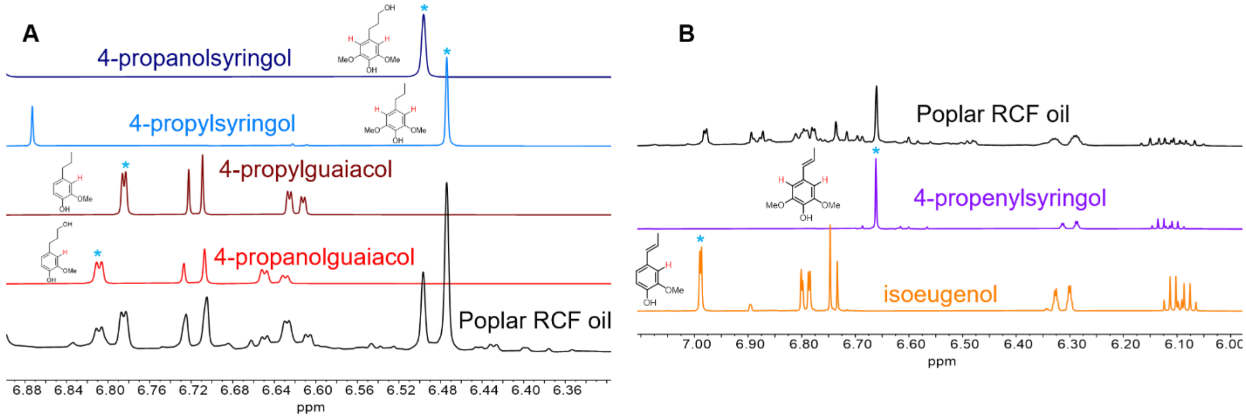

Figure S8. Identification of ^{1}H NMR resonances in poplar. (**A**) Standard RCF reaction with high selectivity to 4-propyl and 4-propanol products. Conditions: 2 grams poplar, 400 mg Ru/C wetted with 400 mg water, 30 mL methanol, 225 °C, 3 hours, 30 bar H_2. (**B**) Example of high selectivity to unsaturated (4-propenyl) side chains through the use of H_2-free conditions. 2 grams poplar, 100 mg Ru/C wetted with 400 mg water, 30 mL methanol, 225 °C, 3 hours, 0 bar H_2.

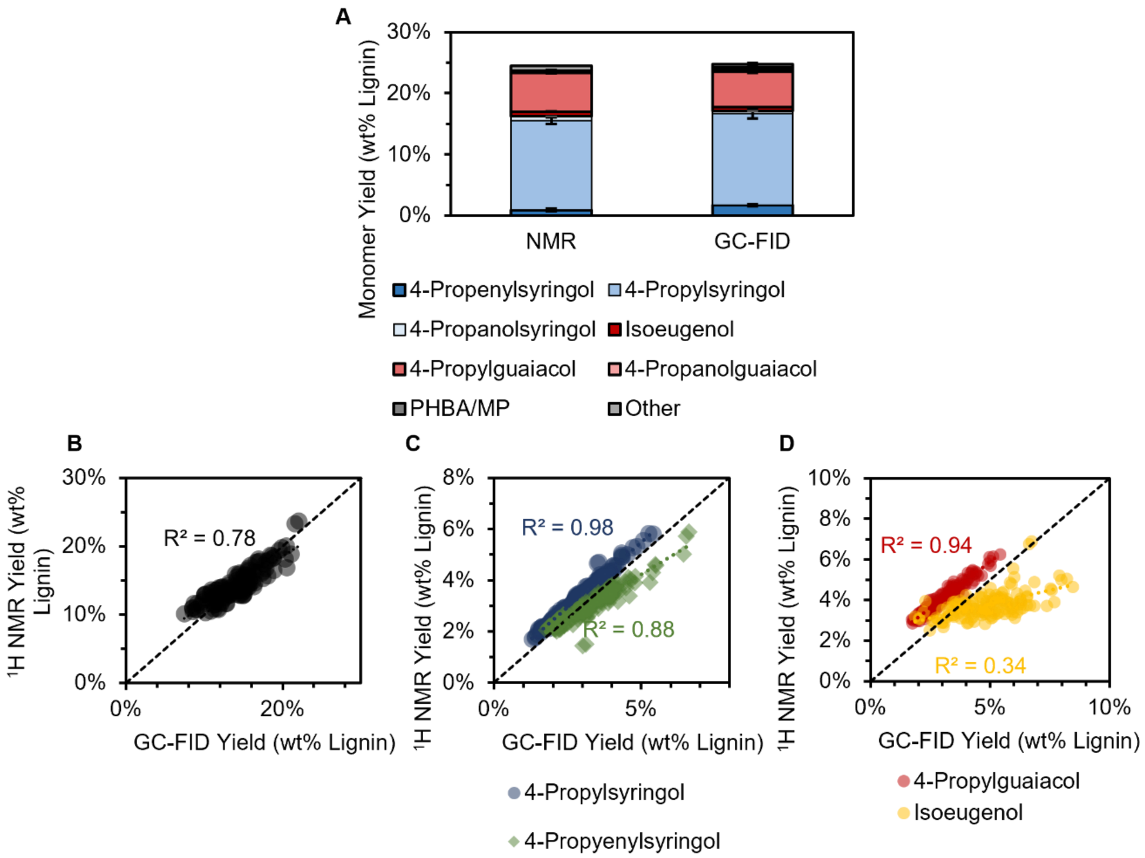

Figure S9. **Calculation of monomer yields from ^{1}H NMR**. Comparison of monomer yields measured by ^{1}H NMR and by GC-FID for (**A**) Conditions: 2 grams poplar, 600 mg 5 wt% Ru/C, 20 mL 1:1 IPA/MeOH, 6 hours, 200 °C. Error bars represent the standard deviation of three measurements. (**B-D**) Parity plots of total (**B**) and individual monomer quantifications of switchgrass samples shown in **Figure 8.**

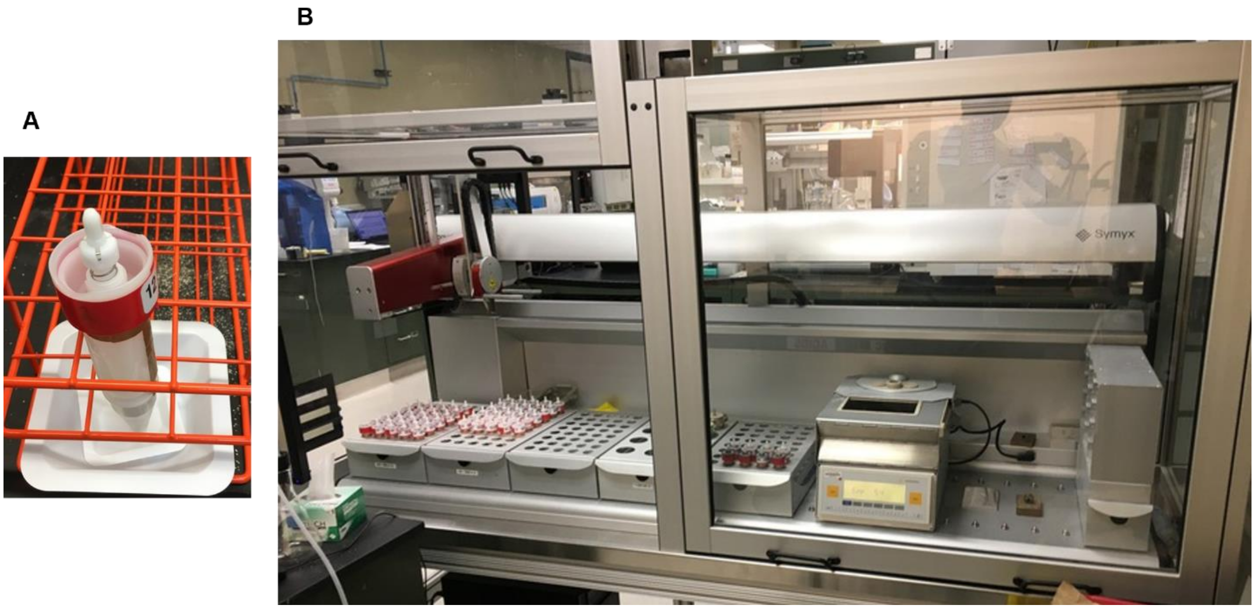

Figure S10. Images of (**A**) a single hopper loaded with biomass (**B**) The solids-loading robot with 70 biomass hoppers loaded.

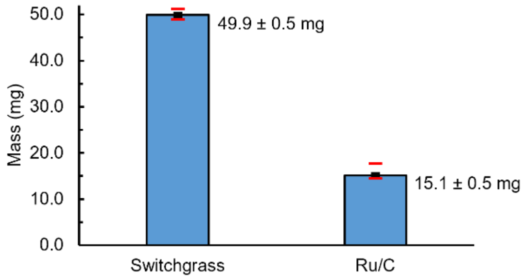

Figure S11. Dispensing accuracy of the Symex Powdernium solids handling robot. The instrument was set to dispense 50 mg of biomass with 1 mg tolerance, and 15 mg of Ru/C with 1 mg tolerance. This data corresponds to the average of 150 dispensing events for switchgrass and Ru/C. Error bars are the standard deviation of all measurements. The red bars indicate the maximum and minimum measurements. Reaction results are shown in **Figure 8**.

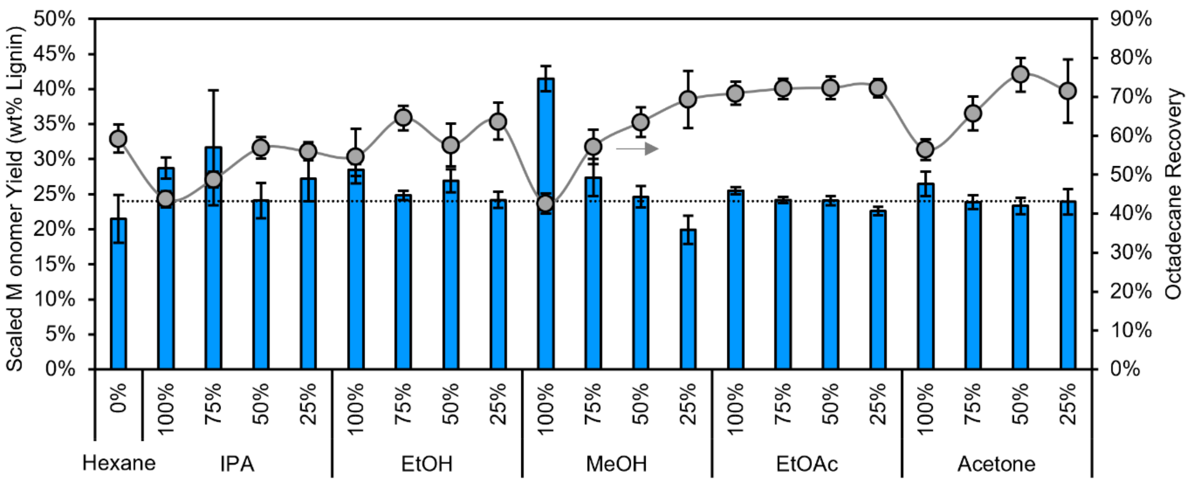

Figure S12: Influence of workup solvent on monomer yield and oil yield. The scaled monomer yields for the solvent combinations tested (blue bars) and octadecane recovery (grey circles) The percentages on the x-axis indicate the volume percent of the listed primary solvent mixed with hexane. The horizontal black line represents the average monomer yield from 75 mL batch reactions.

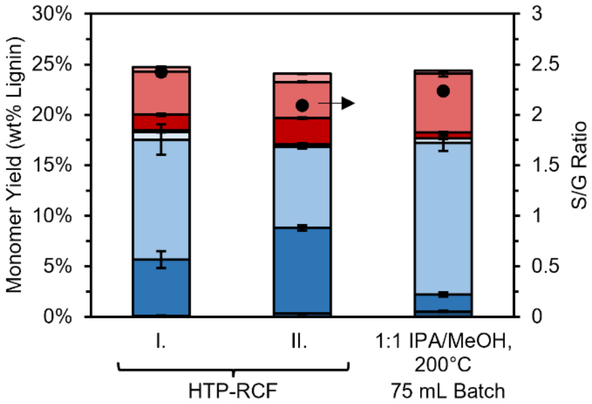

Figure S13. Comparison of monomer yields obtained with different workup methods for HTP-RCF (Method I versus Method II) compared to the 75 mL batch reaction. HTP conditions: 50 mg poplar, 15 mg 5 wt% Ru/C, 0.5 mL 1:1 IPA/MeOH, 6 hours, 200 °C. 75 mL batch conditions: 2 grams poplar, 600 mg 5 wt% Ru/C, 20 mL 1:1 IPA/MeOH, 6 hours, 200 °C. Black dots are the S/G ratiods (right axis). Error bars represent the standard deviation of triplicate measurements for HTP Method I and the 75 mL batch, and twelve measurements for HTP Method II.

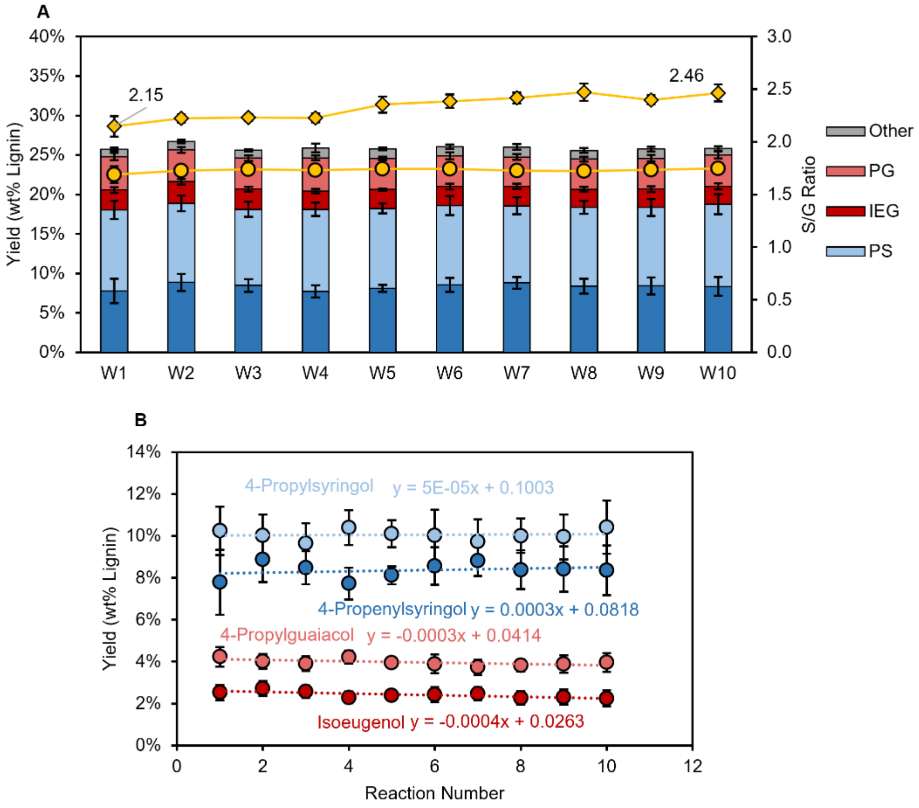

Figure S14. Variation in total monomer yield for control reactions across ten HTP experiments. (**A**) Total monomer yield, and the molar S/G ratio as measured by GC-FID for monomers (yellow diamonds) and by ^{1}H NMR (yellow circles). Each bar is an average of thirty replicate standard reactions run in a single HTP-RCF experiment. The increase measured in GC-FID is absent in the NMR measurement, which indicates the increase in S/G ratio is a result of variation from the GC-FID quantification, rather than from a reaction characteristic. (**B**) GC-FID quantification of individual monomers across the ten reactions, indicating a slight decresase in G-type monomers. Conditions: 50 mg poplar, 15 mg 5 wt% Ru/C, 0.5 mL 1:1 IPA/MeOH, 6 hours, 200 °C, 3:1 EtOAc/hexane wash solvent.

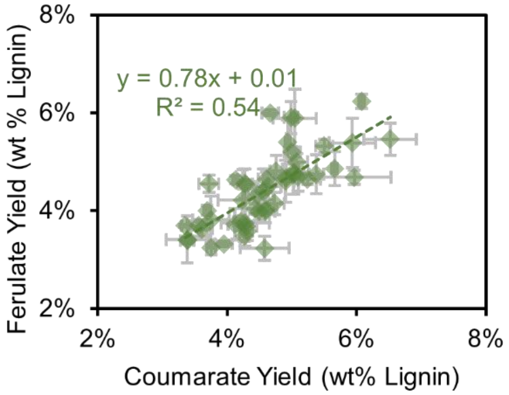

Figure S15. Relationship between coumarate and ferulate derived product yields. Conditions: 50 mg switchgrass, 15 mg Ru/C, 0.5 mL 1:1 IPA/MeOH, 6 h, via workup "Method II". Error bars are the standard deviation of triplicate measurements.

3. SI Tables

Table S1. Equipment list.

Equipment	Purpose	Source	Product Number	Amount per reaction	Amount per 240 sample reaction
Filter Plates, 24 wells	Filter reaction mixture from plates into 4 mL vials	Thomson Scientific	921546	1 per plate	10
Belleville Disc Spring	Compress endplates for plate sealing	McMaster-Carr	9712k64	10 per all thread	120
PTFE O-Rings*	Seal individual wells	McMaster-Carr	9559K19	1	264
Wheaton Sample Vial - 4mL	Capture reaction mixture from filtering	Sigma Aldrich	Z188786	1	240
Wheaton Sample Vial - 4mL caps	Seal 4 mL vials	Sigma Aldrich	Z106429	1	240
GC Vial with 300 µL insert	Inserts allowed lower sample volume without autosampler malfunction	Agilent	5188-6572	1	240
GC Vial Caps	Seal GC vials	Agilent	5183-4498	1	240
Steel Threaded Rod, cut to 14"	Screw into bottom endplate, compress top endplate	McMaster-Carr	99065A216	-	12
Steel Hex Nut	Seal endplates	McMaster-Carr	95462A029	3 per all thread	36
Eclipse™ FlexTop™ 1250µL Wide Orifice Pipet Tips	Transferring of reaction mixture; wide tip is needed to transfer solid material	Thomas Scientific	1042-960-008-9	1	240
Multi-channel pipette	Transfer multiple reactions into filter plate	Rainin	17014497	-	NA
Hastelloy Reactor Plates	Reactors	Aspen Machining Inc. (Lafayette, CO USA)	Custom machined	-	11

***** Several experiments using O-rings constructed of various polymers resulted in total leakage before discovering that PTFE O-rings successfully held pressure with minimal solvent loss.

Table S2. Compositional analysis of poplar used for exploratory experiments. *Populus trichocarpa* genotype GW-9947 is the standard reference poplar of the Center for Bioenergy innovation, and was used as the poplar substrate here for method development.[2] Growth and processing conditions were described previously.[3]

	%Lignin (py-MBMS)*	% Lignin	% Glucan	% Xylan	% Galactan	% Arabinan	% Mannan	% Acetyl	% Other	Total %
GW-9947	23.22	23.80	45.71	15.30	1.17	0.00	3.54	4.01	3.53	97.07

*The lignin measured by pyrolysis with molecular beam mass spectrometry (py-MBMS) was used for calculation of yields throughout this work.[4]

Table S3. RCF data for solvent screening experiments (**Figure 3A**). Conditions: 50 mg poplar, 10 mg 5 wt.% Ru/C, 0.5 mL solvent, 15 h, 180 °C. Analyzed with workup Method I. Values are the average of one point using DMB and one point using TTB as the internal standard (two data points total; each data point pooled three reaction wells). +/- is the range of two data points for total monomer yield.

	PG	PS	EG	ES	IEG	P=S	PG-OH	PS-OH	Oil Yield (Mass)	Oil Yield (NMR)	Monomer Yield	+/-
IPA	0.2%	0.6%	0.1%	3.3%	0.0%	0.0%	0.1%	0.6%	55.6%	7.4%	4.9%	0.6%
7:3 IPA/MeOH	2.1%	4.3%	0.1%	0.0%	0.8%	4.4%	0.1%	0.3%	46.4%	20.0%	12.2%	0.7%
1:1 IPA/MeOH	3.0%	7.0%	0.1%	0.0%	1.4%	4.9%	0.4%	0.5%	57.5%	25.3%	17.3%	1.2%
3:7 IPA/MeOH	2.7%	6.3%	0.1%	0.1%	1.5%	5.2%	0.1%	0.5%	55.6%	25.7%	16.6%	1.3%
MeOH	2.3%	5.5%	0.1%	0.1%	2.1%	6.8%	0.3%	0.5%	52.9%	28.7%	17.9%	0.8%

Table S4. RCF data for time course experiments (**Figure 3B**). Conditions: 50 mg poplar, 10 mg 5 wt% Ru/C, 0.5 mL 1:1 IPA/MeOH, 200 °C. Analyzed with workup Method I. Values are the average of two replicates using DMB and two replicates using TTB as the internal standard (four data points total; each data point pooled three reaction wells). +/- is the standard deviation of four data points for total monomer yield.

Time (hour)	PG	PS	EG	ES	IEG	P=S	PG-OH	PS-OH	Oil Yield (Mass)	Oil Yield (NMR)	Monomer Yield	+/-
1	0.7%	1.1%	0.0%	0.0%	1.3%	2.7%	0.2%	0.1%	35.1%	10.1%	6.0%	0.4%
3	2.0%	4.0%	0.0%	0.1%	2.4%	7.5%	0.6%	0.5%	57.8%	29.4%	17.0%	2.5%
6	2.9%	6.7%	0.0%	0.0%	2.5%	8.6%	0.7%	0.8%	54.3%	33.2%	22.3%	3.9%
15	4.3%	12.3 %	0.1%	0.1%	0.9%	3.6%	0.4%	0.8%	79.8%	36.1%	22.5%	0.7%

Table S5. RCF data for catalyst loading experiments (**Figure 4A**). Conditions: 25-75 mg poplar, 0-15 mg Ru/C, 200 °C, 0.5 mL 1:1 IPA/MeOH, 6 hours reaction time. Analyzed with workup Method II. +/- is the standard deviation of twelve measurements, except for the condition with 50 mg poplar and 15 mg Ru/C which uses measurements from 106 separate wells. Samples were prepared using workup procedure II.

Poplar (mg)	Ru/C (mg)	PG	PS	EG	ES	IEG	P=S	PG-OH	PS-OH	Oil Yield (NMR)	Monomer Yield	+/-
50	0	0.0%	0.0%	0.0%	0.0%	0.0%	0.5%	0.0%	0.0%	24.3%	0.6%	0.2%
50	5	1.1%	1.0%	0.0%	0.1%	4.3%	11.7%	0.8%	0.3%	46.1%	19.4%	0.6%
50	10	2.8%	6.5%	0.0%	0.2%	3.4%	10.0%	0.7%	0.2%	46.1%	23.7%	3.0%
75	15	3.3%	7.1%	0.0%	0.3%	3.2%	10.1%	0.8%	0.4%	47.0%	25.2%	0.9%
50	15	4.3%	10.2%	0.0%	0.3%	2.3%	7.5%	0.6%	0.1%	45.7%	25.3%	2.1%
25	15	6.4%	16.7%	0.0%	0.0%	0.9%	2.3%	0.0%	0.0%	44.7%	26.3%	2.4%

Table S6. RCF data for comparison between HTP-RCF reactions and 75 mL batch reactor (**Figure 4B**). Plate reactor conditions: 50 mg poplar, 15 mg 5 wt. % Ru/C, 0.5 mL 1:1 IPA/MeOH, 6 hours, 200 °C, +/- is the standard deviation of twelve measurements. 75 mL batch conditions: 2 grams poplar, 600 mg 5 wt% Ru/C, 20 mL 1:1 IPA/MeOH, 6 hours, 200 °C. +/- is the standard deviation of triplicate measurements.

		PG	PS	EG	ES	PG=	PS=	PG-OH	PS-OH	Monomer Yield	+/-
Plate reactors	Workup I.	4.3%	11.9%	0.1%	0.1%	1.5%	5.6%	0.4%	0.8%	24.7%	2.0%
	Workup II.	3.6%	8.0%	0.0%	0.3%	2.6%	8.5%	0.8%	0.2%	24.1%	0.4%
75 mL Batch	1:1 IPA/MeOH, 200°C	5.8%	15.0%	0.0%	0.5%	0.6%	1.7%	0.3%	0.5%	24.4%	1.3%
	MeOH, 225°C	7.2%	19.2%	0.0%	0.5%	0.3%	0.8%	1.2%	3.3%	32.5%	0.0%

Table S7. Integral ranges for ferulate and coumarate derived RCF products. All chemical shifts are referenced to the acetone residual solvent peak at δ = 2.05 ppm.

	**Chemical shift (ppm)**	**Structure**
Total S lignin	6.2-6.6	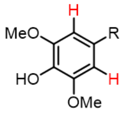<br>R= propyl, propanol, propenyl, or ethyl
Total G lignin	6.6-7.2	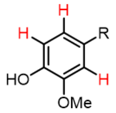<br>R= propyl, propanol, propenyl, or ethyl
Unsaturated hydroxycinnamate α<br>($\mathbf{I_{HC_\alpha} = I_{HC_\beta}}$)	α = 7.57-7.63<br>β = 6.31-6.41	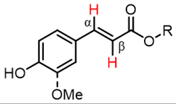
para-Hydroxybenzoic acid	7.91	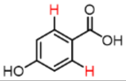
Coumaric acid/methyl coumarate	7.53-7.56	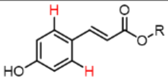
Ferulic acid/methylferulate	7.33	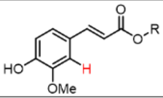
Dihydrocoumaric acid<br>3-(4-hydroxyphenyl)propionic acid	7.03-7.06	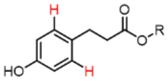
Methylhydrocoumarate<br>methyl 3-(4-hydroxyphenyl)propanoate	7.07	
Hydroferulic acid<br>3-(4-hydroxy-3-methoxyphenyl)propionic acid	6.87	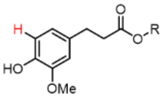
Methylhydroferulate<br>methyl 3-(4-hydroxy-3-methoxyphenyl)propanoate	6.84	

Table S8. Comparison of the gravimetric RCF oil mass with the quantification from the ^{1}H NMR method for a range of oil concentrations (**Figure 5C**).

	Variant (Monomer S/G Ratio)	Gravimetric Mass (mg)	^{1}H NMR Mass (mg)
Poplar	BESC-904 (2.12)	13.1	14.2
		6.5	5.7
		2.6	2.6
	BEC-64 (1.82)	11.4	10.9
		5.7	5.7
		2.3	2.0
	BESC-79 (2.08)	12.6	12.2
		6.3	6.4
		2.5	2.6
	BESC-30 (2.01)	5.7	6.4
		2.3	2.3
	INL-2021 (1.29)	7.7	7.2
		14.7	13.4
		22.8	20.3
		36.4	30.2
Pine	Pine	12.6	14.1
		6.3	5.4
		2.5	1.9
Switchgrass	20TIF.J222.A.CV.B	36.8	18.5
		15.0	10.1
		6.8	5.3
	20UTK.J466.B.Low_N.B	37.1	14.3
		14.2	8.0
		7.7	4.4
	20IHF.J041.A.P6.B	33.8	16.9
		14.6	10.9
		7.7	5.4

Table S9. Results of wash solvent screening. A 75% mix with hexane represents a mixture that is 75% primary solvent and 25% hexane by volume. The 75% EtOAc wash solvent selected for further use is bolded. CV is the coefficient of variation, defined as CV = sample standard deviation/sample average

	Solvent % Mix with Hexane	C18 Recovery	±	Unscaled Monomer Yield	±	CV	Scaled Monomer Yield	±	CV
Hexane	0%	59.3%	3.6%	12.7%	2.2%	17.3%	21.5%	3.4%	15.9%
IPA	100%	43.7%	2.1%	12.5%	0.8%	6.1%	28.7%	1.5%	5.3%
	75%	48.8%	2.1%	15.4%	3.8%	24.6%	31.6%	8.2%	25.9%
	50%	56.9%	2.7%	13.7%	1.4%	10.1%	24.1%	2.5%	10.5%
	25%	56.0%	2.3%	15.3%	2.5%	16.1%	27.2%	3.2%	11.6%
EtOH	100%	54.8%	7.0%	15.6%	1.9%	12.3%	28.5%	0.9%	3.0%
	75%	64.6%	3.1%	16.0%	0.6%	3.7%	24.8%	0.7%	2.6%
	50%	57.7%	5.5%	15.5%	1.0%	6.8%	26.9%	1.6%	6.0%
	25%	63.7%	4.7%	15.4%	1.2%	7.5%	24.2%	1.2%	4.8%
MeOH	100%	42.6%	2.6%	17.7%	0.9%	5.1%	41.5%	1.8%	4.3%
	75%	57.2%	4.4%	15.6%	1.4%	9.0%	27.4%	2.6%	9.6%
	50%	63.5%	3.8%	15.6%	0.8%	5.4%	24.6%	1.5%	6.2%
	25%	69.3%	7.4%	13.7%	1.1%	7.8%	19.9%	2.0%	10.1%
EtOAc	100%	70.9%	3.0%	18.1%	0.8%	4.3%	25.5%	0.5%	2.0%
	**75%**	**72.1%**	**2.6%**	**17.4%**	**0.6%**	**3.4%**	**24.1%**	**0.4%**	**1.8%**
	50%	72.3%	2.8%	17.4%	0.8%	4.3%	24.1%	0.7%	2.8%
	25%	72.2%	2.4%	16.3%	0.8%	4.9%	22.6%	0.6%	2.6%
Acetone	100%	56.5%	2.7%	15.0%	1.0%	6.8%	26.5%	1.8%	6.6%
	75%	65.7%	4.4%	15.7%	1.3%	8.5%	23.9%	1.0%	4.0%
	50%	75.7%	4.3%	17.6%	0.9%	5.0%	23.3%	1.2%	4.9%
	25%	71.5%	8.2%	17.0%	1.2%	7.1%	23.9%	1.8%	7.6%

Table S10. Results of switchgrass screening. Abbreviations: 4-ethylphenol, EP; 4-Ethylguaiacol, EG; 4-Propylguaiacol, PG; Isoeugenol, IEG; 4-Propylsyringol, PS; 4-Propanolguaiacol, PG-OH; 4-Propenylsyringol, P=S; 4-Propanolsyringol, PS-OH; Products derived from *p*-coumaric acid groups (CA), products deriving from ferulic acid groups (FA). Total is the sum of all phenolic monomers including CA and FA.

Genotype	EP	EG	PG	IEG	PS	PG-OH	P=S	PS-OH	CA	FA	Total	+/-	Oil	+/-	S/G	+/-
J222.A.CV.B	2.5%	0.6%	4.8%	6.4%	3.6%	0.5%	6.5%	0.0%	6.0%	4.7%	35.5%	1.4%	75.3%	4.3%	0.73	0.04
J191.A.F8	3.2%	0.6%	4.2%	5.1%	4.6%	1.2%	4.0%	0.0%	5.9%	5.4%	34.2%	1.3%	62.8%	1.6%	0.70	0.02
J008.D.L34	3.7%	0.6%	4.2%	4.0%	5.3%	0.0%	3.3%	0.5%	6.1%	6.2%	33.8%	0.4%	61.6%	0.9%	0.93	0.02
J295.A.UC.B	2.2%	0.1%	3.8%	8.0%	3.4%	0.3%	5.4%	0.0%	4.6%	3.2%	31.0%	0.2%	55.0%	2.8%	0.62	0.02
AP13.C23	2.6%	0.5%	4.5%	5.9%	3.8%	0.4%	4.7%	0.0%	4.2%	3.8%	30.3%	0.9%	57.5%	1.2%	0.67	0.03
J222.A.UC.B	2.7%	0.0%	5.1%	5.4%	4.5%	0.5%	4.1%	0.0%	4.3%	3.5%	30.0%	0.8%	54.3%	1.2%	0.66	0.00
J317.A.A15	2.9%	0.4%	3.8%	4.0%	4.2%	1.1%	3.6%	0.5%	4.6%	4.6%	29.7%	0.3%	52.4%	1.1%	0.79	0.02
J339.A.B2	3.0%	0.7%	3.3%	5.7%	3.2%	0.8%	3.8%	0.1%	4.6%	4.4%	29.6%	1.5%	56.0%	1.6%	0.63	0.06
J594.A.L42.B	2.7%	0.5%	3.7%	3.7%	3.9%	1.3%	3.0%	0.3%	5.7%	4.9%	29.6%	0.9%	53.8%	0.6%	0.71	0.02
J305.A.R44.B	2.1%	0.5%	2.9%	4.5%	3.7%	0.9%	4.5%	0.1%	5.0%	5.2%	29.5%	2.0%	53.2%	3.0%	0.85	0.07
J499.B.J18.B	2.8%	0.5%	3.0%	5.7%	3.2%	0.4%	3.3%	0.5%	5.0%	4.7%	29.2%	1.0%	51.2%	1.4%	0.65	0.05
J295.A.CV.B	1.6%	0.4%	3.4%	7.8%	2.9%	0.4%	4.0%	0.0%	4.6%	4.0%	29.1%	0.2%	54.6%	0.3%	0.50	0.00
B6.D6	3.0%	0.4%	2.8%	3.5%	3.1%	0.4%	2.7%	1.0%	6.5%	5.5%	29.1%	0.7%	61.0%	2.6%	0.87	0.09
PTC-7.N44	2.2%	0.4%	4.1%	4.5%	3.7%	0.7%	3.3%	0.0%	5.2%	4.6%	28.9%	1.4%	53.3%	1.8%	0.65	0.08
J301.A.B14.B	2.7%	0.4%	3.0%	4.0%	3.6%	1.0%	3.3%	0.0%	5.4%	4.7%	28.3%	1.7%	50.9%	1.7%	0.74	0.09
J177.A.M45	2.5%	0.6%	2.5%	5.1%	2.9%	0.9%	4.0%	0.1%	4.8%	4.8%	28.2%	2.1%	55.0%	0.1%	0.71	0.07
J218.A.S23.B	2.8%	0.5%	2.7%	4.9%	3.5%	0.5%	4.6%	0.0%	4.4%	4.0%	27.9%	0.6%	51.8%	0.4%	0.85	0.05
J212.A.L18.B	1.9%	0.3%	3.7%	5.5%	2.8%	0.6%	3.6%	0.0%	4.7%	4.1%	27.3%	1.9%	53.5%	1.2%	0.57	0.05
J496.C.C37	2.5%	0.5%	3.0%	2.6%	3.8%	1.7%	2.4%	0.8%	5.0%	4.8%	27.2%	1.7%	51.9%	1.8%	0.82	0.04
J041.A.P6	2.5%	0.5%	2.4%	6.3%	2.6%	0.3%	4.4%	0.0%	4.3%	3.7%	27.0%	0.3%	52.2%	3.2%	0.67	0.07
J294.A.M7.B	2.6%	0.5%	2.7%	4.0%	3.0%	1.0%	2.9%	0.3%	5.1%	4.7%	27.0%	0.9%	49.0%	0.8%	0.69	0.07
J297.A.D34.B	2.6%	0.4%	2.6%	5.1%	3.1%	0.6%	4.0%	0.0%	4.5%	4.0%	27.0%	0.6%	50.0%	0.5%	0.74	0.01
J296.A.G37	3.1%	0.5%	2.4%	4.9%	3.0%	0.7%	3.6%	0.3%	4.6%	4.0%	26.9%	1.3%	47.6%	1.2%	0.74	0.10
J311.A.UC.B	1.6%	0.4%	3.3%	7.2%	3.1%	0.2%	3.7%	0.0%	3.6%	3.7%	26.7%	1.0%	47.8%	1.1%	0.54	0.00
J022.A.F20	2.8%	0.4%	2.6%	5.1%	3.3%	0.6%	4.0%	0.0%	4.3%	3.6%	26.6%	0.5%	47.4%	0.4%	0.74	0.01
J211.A.K41	1.9%	0.6%	3.5%	6.2%	2.4%	0.7%	2.6%	0.0%	4.2%	4.2%	26.5%	1.3%	49.5%	3.9%	0.41	0.00
J226.A_LowN_D4	2.4%	0.4%	2.2%	4.2%	2.8%	0.5%	3.8%	0.6%	4.9%	4.7%	26.5%	0.5%	49.2%	0.8%	0.89	0.10
J226.A_ModN_X4	2.2%	0.4%	2.2%	4.1%	2.8%	0.7%	3.9%	0.5%	4.9%	4.6%	26.4%	0.4%	49.4%	1.2%	0.89	0.06
J321.A.C13	2.4%	0.4%	3.2%	3.0%	3.2%	1.5%	2.0%	0.4%	5.1%	5.0%	26.3%	0.3%	45.8%	0.9%	0.62	0.02
J331.A.H20	2.1%	0.5%	4.4%	5.2%	3.3%	0.6%	3.0%	0.0%	3.4%	3.7%	26.1%	0.6%	45.4%	0.3%	0.53	0.04

J324.A.G29	2.1%	0.5%	3.1%	5.1%	2.3%	0.7%	3.2%	0.0%	4.3%	4.5%	25.8%	0.1%	47.6%	0.5%	0.52	0.00
J323.A.F18	2.2%	0.5%	3.7%	5.8%	2.5%	0.6%	3.1%	0.0%	3.6%	3.6%	25.7%	0.7%	47.8%	2.5%	0.48	0.04
J615.A.B20	1.6%	0.5%	2.7%	3.3%	2.3%	1.1%	2.3%	0.7%	5.0%	5.9%	25.4%	1.7%	51.2%	1.3%	0.66	0.13
J484.B.A9	2.3%	0.6%	2.8%	5.6%	2.8%	0.4%	3.6%	0.3%	3.8%	3.2%	25.2%	0.4%	47.7%	0.2%	0.64	0.03
J303.A.A23.B	1.7%	0.6%	2.9%	3.7%	2.1%	1.2%	2.2%	0.3%	4.9%	5.4%	25.1%	1.2%	51.0%	0.7%	0.51	0.04
J009.C.L10	1.9%	0.5%	2.3%	6.1%	2.4%	0.4%	4.1%	0.0%	3.9%	3.3%	25.0%	0.7%	48.5%	1.3%	0.63	0.01
J311.A.CV.B	1.7%	0.5%	3.3%	3.4%	2.8%	0.0%	2.2%	0.0%	5.5%	5.3%	24.6%	2.6%	47.6%	0.6%	0.68	0.19
J497.C.E27	1.6%	0.5%	2.4%	3.5%	2.3%	0.5%	2.1%	1.0%	4.7%	6.0%	24.6%	1.3%	47.6%	0.7%	0.71	0.10
J521.B.H40	2.0%	0.5%	3.2%	5.8%	2.0%	0.5%	2.6%	0.0%	4.3%	3.7%	24.6%	1.1%	47.8%	2.3%	0.41	0.04
J251.C.G23.B	1.9%	0.5%	2.2%	5.4%	2.0%	0.5%	3.4%	0.0%	4.5%	4.3%	24.6%	0.3%	47.6%	1.2%	0.58	0.04
J215.A.G41.B	1.4%	0.5%	2.3%	6.0%	1.6%	0.5%	2.8%	0.2%	4.6%	4.5%	24.5%	0.7%	50.6%	2.0%	0.44	0.04
J073.B.H12	2.3%	0.5%	2.5%	4.5%	2.7%	0.7%	3.2%	0.1%	3.7%	3.8%	23.9%	1.8%	43.8%	1.3%	0.66	0.04
J463.A.D14	1.9%	0.6%	2.4%	5.5%	1.7%	0.6%	2.8%	0.1%	4.1%	3.7%	23.4%	1.5%	45.6%	0.4%	0.47	0.05
J466.B_ModN_DD31	1.6%	0.6%	1.8%	3.5%	1.6%	0.5%	2.0%	0.8%	5.0%	5.9%	23.2%	1.9%	48.5%	2.3%	0.63	0.08
J312.A.F24	1.3%	0.4%	3.1%	5.5%	2.3%	0.6%	3.1%	0.0%	3.4%	3.4%	23.1%	0.3%	45.2%	0.5%	0.50	0.03
J504.C_LowN_I17	1.7%	0.4%	2.6%	4.1%	1.6%	0.7%	2.3%	0.0%	4.3%	4.5%	22.3%	2.1%	43.2%	0.3%	0.49	0.18
J073.A.F4	2.1%	0.5%	1.8%	4.7%	2.0%	0.4%	3.1%	0.0%	3.4%	3.4%	21.4%	0.9%	41.7%	2.3%	0.62	0.05
J504.C_ModN_CC17	1.7%	0.0%	2.8%	3.9%	1.8%	1.1%	2.3%	0.0%	3.7%	4.0%	21.1%	1.2%	40.8%	0.4%	0.45	0.10
J587.B.M27	1.3%	0.5%	2.2%	2.8%	2.0%	0.9%	1.8%	0.6%	4.1%	4.6%	20.9%	1.2%	42.4%	0.6%	0.64	0.12
J466.B_LowN_J31	1.3%	0.5%	2.1%	3.9%	1.4%	1.1%	2.1%	0.3%	3.7%	4.5%	20.9%	2.3%	40.0%	1.0%	0.47	0.11

Table S11. Comparison of yields from HTP-RCF and 75 mL batch reactions.

		PG	PS	PG-OH	Ps-OH	IEG	P=S	EP	CA	FA	Other	S/G	Oil	Total
J222.A.CV.B	Parr	6.5%	7.1%	2.2%	3.0%	0.3%	0.3%	1.4%	6.5%	3.9%	1.2%	0.95	59.7%	32.3%
	HT	4.8%	3.6%	0.5%	0.0%	6.4%	6.5%	2.5%	6.0%	4.7%	0.6%	0.73	75.3%	35.5%
J041.A.P6.B	Parr	4.3%	5.1%	1.8%	3.1%	0.7%	0.7%	1.7%	5.9%	4.0%	1.1%	1.05	51.4%	28.4%
	HT	2.4%	2.6%	0.3%	0.0%	6.3%	4.4%	2.5%	4.3%	3.7%	0.5%	0.67	52.2%	27.0%
J296.A.G37.B	Parr	4.1%	5.3%	0.9%	1.6%	2.3%	0.5%	2.1%	5.7%	4.0%	0.5%	0.81	50.6%	27.0%
	HT	2.4%	3.0%	0.7%	0.3%	4.9%	3.6%	3.1%	4.6%	4.0%	0.5%	0.74	47.6%	26.9%
J324.A.G29.B	Parr	5.0%	4.0%	1.9%	2.0%	1.8%	0.8%	1.2%	5.0%	4.4%	0.4%	0.62	56.4%	26.3%
	HT	3.1%	2.3%	0.7%	0.0%	5.1%	3.2%	2.1%	4.3%	4.5%	0.5%	0.52	47.6%	25.8%
J521.B.H40.B	Parr	5.1%	3.5%	1.7%	1.6%	2.2%	0.7%	1.2%	5.2%	4.3%	0.4%	0.53	55.3%	26.0%
	HT	3.2%	2.0%	0.5%	0.0%	5.8%	2.6%	2.0%	4.3%	3.7%	0.5%	0.41	47.8%	24.6%
J466.B.Low_N.B	Parr	3.5%	2.7%	1.6%	1.9%	0.7%	0.4%	1.1%	3.8%	4.4%	1.3%	0.66	44.8%	21.4%
	HT	2.1%	1.4%	1.1%	0.3%	3.9%	2.1%	1.3%	3.7%	4.5%	0.5%	0.47	40.0%	20.9%

Table S12. Comparison of material and time requirements for conventional and batch RCF.

	75 mL batch	HTP
Samples	50	
Replicates	3	
Total reactions	150	
Ru/C per reaction (g)	0.4	0.015
**total Ru/C (g)**	60	2.25
Solvent per reaction (mL)	30	0.5
**total solvent (mL)**	4500	75
Biomass per reaction (g)	2	0.05
**total biomass (g)**	300	7.5
Reactions per set	6	240
Sets required	25	0.625
Time per set: solids loading, solvent addition, sealing (hour)	1	6
Time per set: filtration, evaporation (hour)	6	6
Time per set: sample preparation (hour)	1	6
Time per set: sample analysis (hour)	0.5	4
**Sum (hour)**	8.5	22
Time per reaction (hour/reaction)	1.42	0.092
**Tiem Ratio: 75 mL batch/HTP**		15.45

(1) Happs, R. M.; Hanes, R. J.; Bartling, A. W.; Field, J. L.; Harman-Ware, A. E.; Clark, R. J.; Pendergast, T. H.; Devos, K. M.; Webb, E. G.; Missaoui, A.; Xu, Y.; Makaju, S.; Shrestha, V.; Mazarei, M.; Stewart, C. N.; Millwood, R. J.; Davison, B. H. Economic and Sustainability Impacts of Yield and Composition Variation in Bioenergy Crops: Switchgrass (*Panicum Virgatum* L.). *ACS Sustainable Chem. Eng.* **2024**, acssuschemeng.3c05770.

(2) Biswal, A. K.; Hengge, N. N.; Black, I. M.; Atmodjo, M. A.; Mohanty, S. S.; Ryno, D.; Himmel, M. E.; Azadi, P.; Bomble, Y. J.; Mohnen, D. Composition and Yield of Non-Cellulosic and Cellulosic Sugars in Soluble and Particulate Fractions during Consolidated Bioprocessing of Poplar Biomass by Clostridium Thermocellum. *Biotechnol Biofuels* **2022**, *15* (1), 23.

(3) Happs, R. M.; Bartling, A. W.; Doeppke, C.; Harman- Ware, A. E.; Clark, R.; Webb, E. G.; Biddy, M. J.; Chen, J.; Tuskan, G. A.; Davis, M. F.; Muchero, W.; Davison, B. H. Economic Impact of Yield and Composition Variation in Bioenergy Crops: *Populus Trichocarpa*. *Biofuels Bioprod Bioref* **2021**, *15* (1), 176–188.

(4) Harman-Ware, A. E.; Macaya-Sanz, D.; Abeyratne, C. R.; Doeppke, C.; Haiby, K.; Tuskan, G. A.; Stanton, B.; DiFazio, S. P.; Davis, M. F. Accurate Determination of Genotypic Variance of Cell Wall Characteristics of a Populus Trichocarpa Pedigree Using High-Throughput Pyrolysis-Molecular Beam Mass Spectrometry. *Biotechnol Biofuels* **2021**, *14* (1), 59.
